# Physical viability of the massive pulsar PSR J0952–0607 within a polytropic framework

**DOI:** 10.1371/journal.pone.0359628

**Published:** 2026-10-07

**Authors:** Muhammad Bilal Riaz, A. Zahra, S. A. Mardan

**Affiliations:** 1 IT4Innovations, VSB – Technical University of Ostrava, Ostrava, Czech Republic; 2 Department of Mathematics, Firat University, Elazig, Turkiye; 3 Department of Mathematics, University of Management and Technology, Lahore, Pakistan; 4 Center for Theoretical Physics, Khazar University, Baku, Azerbaijan; Yibin University, CHINA

## Abstract

We develop a relativistic model for one of the most massive pulsars, PSR J0952-0607, by constructing exact solutions of Einstein’s field equations (EFEs) within the framework of the generalized Tolman–Kuchowicz (GTK) spacetime. The stellar interior is modeled as anisotropic matter governed by a polytropic equation of state (EoS). This framework allows for a systematic investigation of how the GTK metric exponent *m* and the polytropic index *n* influence the internal structure and stability of the star. The physical characteristics of the model are analyzed using three-dimensional graphical representations. These plots simultaneously display the dependence of key variables on the radial coordinate as well as on the model parameters *m* and *n*. By smoothly matching the interior solution to the exterior Schwarzschild spacetime, the model parameters are constrained using the measured mass $M=2.35\pm 0.17\;M_{⊙}$ and radius $13.7_{-1.5}^{+2.6}\;km$ estimates of PSR J0952-0607. We analyze the radial behavior of the energy density, radial and tangential pressures, anisotropy, sound speeds, adiabatic indices, and hydrostatic equilibrium through the Tolman–Oppenheimer–Volkoff (TOV) equation. All physical quantities remain finite, positive and well behaved, while the causality condition, Herrera’s cracking criterion, and the relativistic stability requirement Γ>4/3 are satisfied throughout the stellar interior. The model fulfills all standard energy conditions and yields mass and compactness profiles compatible with representative NICER-based constraints for massive neutron stars. This shows the physical viability of anisotropic polytropic configurations for ultra-massive neutron stars.

## 1. Introduction

The general theory of relativity (GR) has repeatedly confirmed its reliability by accurately describing numerous astrophysical phenomena. It has played a crucial role in explaining the precession of Mercury’s orbit and, more recently, in the detection of gravitational waves generated by merging black holes, underscoring its relevance in astrophysics [1]. Stars originate within immense interstellar clouds of gas and dust scattered throughout galaxies. As massive stars evolve and near the end of their life cycle, they develop dense cores. At a critical point, the pressure of nuclear fusion is no longer sufficient to counteract gravitational forces. When the star loses its equilibrium state, it collapses under its own gravity, which is known as stellar collapse [2]. The outcome of gravitational collapse is a compact stellar remnant, such as a white dwarf, neutron star, black hole, or quark star, determined by the star’s initial mass. These compact remnants exist in strong gravitational fields where relativistic effects become significant, which requires analysis through EFEs. Due to their complexity, being non-linear and highly coupled partial differential equations, obtaining exact solutions to EFEs is a formidable challenge. Nevertheless, some exact and physically meaningful solutions have been discovered. Among the earliest was the Schwarzschild solution, which describes both the interior [3] and exterior [4] gravitational fields of a static, spherically symmetric mass with uniform density. In a survey of 127 EFE solutions conducted by Delgaty and Lake [5], only 16 were found to meet all criteria for physical acceptability, and just 9 of these featured a sound speed that decreased with increasing radius.

A pulsar is a neutron star that spins rapidly and emits radiation beams from its magnetic poles, appearing as regular pulses when seen from Earth. They are important because they serve as extremely precise cosmic clocks, help test Einstein’s theory of GR, and aid in the study of the galaxy and gravitational waves. Within our Galaxy, PSR J0952–0607 is identified as an ultra-massive pulsar. It’s part of a binary system and is known for being one of the fastest-spinning neutron stars ever discovered. Observations indicate that it is among the most massive neutron stars known, with an estimated mass of approximately $2.35\pm 0.17\;M_{⊙}$. Because of this extreme mass, the pulsar serves as an important observational probe to place limits on the neutron star EoS. The radius $R=13.7_{-1.5}^{+2.6}\;km$ is not a direct measurement of PSR J0952-0607; it was obtained for PSR J0740 + 6620 from NICER and XMM–Newton observations [6]. The millisecond pulsar PSR J0952–0607 was first reported by Bassa et al. [7] as the fastest such object detected in the Galactic field.

They examined that the pulsars exhibit unusually steep radio spectra and explored the potential for discovering additional sources similar to PSR J0952-0607. Romani et al. [8] explored the observations of PSR J0952-0607 and identified it as the fastest spinning known neutron star, with a precisely calculated mass of $2.35\pm 0.17\;M_{⊙}$ the highest mass determined to date. The findings suggest that it has accreted nearly $1\;M_{⊙}$, and set a lower limit on the highest mass of a neutron star at $M_{\text{max}}>2.19\;M_{⊙}$ with 1σ confidence. Kumar et al. [9] studied the constraint of EoS for dense matter using the maximum mass of PSR J0952-0607. Three new relativistic mean-field models are proposed, which align with nuclear-matter characteristics and astrophysical observations, predicting a neutron star radius of 12.98−13.09 km and satisfying constraints from the NICER and GW170817 data. Wu et al. [10] investigated the PSR J0952-0607 pulsar to improve the limits of neutron star EoS, emphasizing the importance of rotational effects. Nieder et al. [11] studied about the PSR J0952 0607 which was previously found in search of gamma-ray sources as a result of LOFAR.

In the context of compact stellar objects, pressure anisotropy emerges when the stresses acting along the radial direction differ from those acting tangentially. This effect can be measured through the anisotropy parameter $\Delta =p_{t}-p_{r}$. This anisotropic behavior could be caused by mechanisms such as phase transitions in dense matter, strong magnetic fields, or superfluid components, and the internal structure, stability, and maximum mass of compact stars should be established. Becerra et al. [12] in their study of anisotropic neutron stars with the help of the Hartle-Thorne formalism, demonstrated that anisotropy has a significant impact on the properties of the star and that it can raise the maximum mass by up to 60% of that of a star of isotropic nature. Becerra et al. [13] investigated the role of pressure anisotropy in neutron stars modeled with piecewise polytropic EoS, showing that anisotropic effects can significantly increase the attainable maximum mass. Their results suggest that such enhancements may help to explain the formation of exceptionally massive compact objects. Kumar and Bharti [14] analyzed compact anisotropic stars within a relativistic framework and demonstrated that anisotropy is important in shaping the internal stellar structure. Hossien et al. [15] discussed the emergence of anisotropic stellar configurations induced by a cosmological term that is dependent on radius within the Karmarkar–Krori–Barua geometry.

The model satisfies all regularity, stability, and physical conditions and is demonstrated with 4U 1820−30 as a representative example. Ruderman [16] discussed that at sufficiently high densities, nuclear matter can develop anisotropic pressure distributions. Subsequently, a number of studies [17–25] have constructed exact solutions of EFEs that provide physically acceptable models for the interior gravitational fields of anisotropic fluid configurations.

An EoS describes the relation between pressure and energy density inside a stellar structure. It describes how matter behaves under conditions of extreme density and pressure. In the study of compact objects, EoS is important for determining the mass–radius relation and assessing the physical viability of stellar models. Ngubelanga [26] studied exact solutions for anisotropic charged compact objects with linear EoS. The Waidya–Tikekar model explored by Sharma et al. [27] using linear EoS. They obtained exact solutions for anisotropic stellar configurations and examined the viability of model.

Vertogradov [28] investigated gravitational collapse within the generalized Vaidya spacetime using polytropic and generalized polytropic EoS, deriving exact mass functions by solving the Einstein and Einstein–Maxwell equations. His findings reveal that the collapse can result in black holes or naked singularities, with the emergence of the latter unaffected by the polytropic index. Berbel and Serna [29] proposed a piecewise polytropic model to more effectively describe phase transitions in neutron star EoS. Maharaj and Matondo [30] proposed new exact solutions to the EFEs of uncharged, anisotropic matter with a generalized polytropic EoS, which contains polytropic and quark matter models as special cases. Hernandez et al. [31] proposed an algorithm to build anisotropic models based on barotropic EoS and introduce a particular assumption on the metric functions, building two physically acceptable models with varying polytropic indices. This generalized method avoids the unphysical behaviors of polytropic indices being greater than one, as in standard anisotropic polytropic models. Singh et al. [32] studied anisotropic stars using a modified polytropic EoS within Korkina-Orlyanskii spacetime, analyzing four models with different polytropic indices.

The GTK is a generalized spacetime that is used to describe anisotropic compact stars, in which the radial and tangential pressures are different. It enables the precise solutions of EFEs with intricate EoS, such as polytropic or linear. These metric potentials facilitate plausible stellar models that meet physical acceptability criteria such as energy, causality, and stability conditions. Additionally, models constructed with the help of the GTK are compatible with observational data, such as mass-radius correlations and tidal deformability. Biswas et al. [33] with the use of the Tolman-Kuchowicz (TK) metric developed a gravitational model of anisotropic strange stars. To make sure that the model was physically stable and plausible, their model was put through multiple physical viability tests, such as the density and pressure being finite and well-behaved. Building on the TK geometry, Jasim et al. [34] proposed another strange-star model that incorporated the MIT bag model for quark matter along with a radially varying cosmological constant. Bhar et al. [35] used the TK spacetime to study compact objects in Einstein–Gauss–Bonnet gravity. Their results demonstrated that, in comparison to GR, the coupling constant has a substantial impact on the stability and stiffness of the model. In order to provide a more flexible description of the interior geometry of the compact star PSR J0952–0607, Acharya et al. [36] computed the EFEs using GTK metric potentials. They developed stable and physically realistic star configurations using a quadratic EoS. To establish the physical viability of the model, the interior solutions of the EFEs must satisfy the regularity conditions specified in [37].

To study the stellar structure of PSR J0952–0607, it is important to gain a deeper understanding of its matter composition and strong gravitational field. These astrophysical objects serve as natural laboratories for testing theoretical models under extreme conditions that are unattainable on Earth. Traditional EoS often fail to accurately model the complexities observed in such dense environments, motivating our use of a polytropic EoS combined with a GTK spacetime. By developing precise and physically viable solutions to Einstein’s equations that satisfy all the stability and causality criteria. This article is organized as follows. Section 2, discusses the solution to EFEs. Section 3 describes the EFEs using the GTK metric. In Section 4, we discuss the boundary conditions and determine the parametric values. Section 5 presents a detailed physical analysis of our model. Finally, in Section 6, we summarize our results.

## 2. Derivation of the EFE solutions

We assumed a static line element in the spherically symmetric spacetime, which is expressed as


$$\begin{array}{c} ds^{2}=e^{\nu (r)}dt^{2}-r^{2}(d\theta^{2}+sin^{2}\theta d\phi^{2})-e^{\lambda (r)}dr^{2}. \end{array}$$ (1)

The GTK metric can be expressed as


$$\begin{array}{c} e^{\lambda }=(1+ar^{2}+br^{4}{)}^{m},\;e^{\nu }=A^{2}e^{Br^{2}}. \end{array}$$ (2)

Here, the parameter *m* is a positive real constant constrained by m≥1 [38]. For *m* = 1, the metric reduces to the standard TK form reported in [39,40]. The constants *a*, *b*, *A*, and *B* remain free parameters and are determined by imposing appropriate boundary conditions. The exponent *m* therefore determines how rapidly the radial metric component changes from the center toward the stellar surface. A larger value of *m* produces a stronger radial deformation of the interior spacetime. Although *m* is not a thermodynamic variable such as temperature, chemical potential, or particle fraction, it has a direct mechanical interpretation because the spacetime geometry determines the matter variables through EFEs. The EFEs can be written as


$$\begin{array}{c} R_{jk}-\frac{1}{2}g_{jk}R=8\pi T_{jk}. \end{array}$$ (3)

For a static spherically symmetric configuration with anisotropic matter content, the energy–momentum tensor is taken as $T_{jk}=diag(-\rho ,p_{r},p_{t},p_{t})$.

The EFEs are expressed as


$$\begin{array}{cc} 8\pi \rho & =e^{-\lambda }(\frac{r\lambda^{\prime }}{r^{2}}-\frac{1}{r^{2}})+\frac{1}{r^{2}}, \end{array}$$ (4)

$$\begin{array}{cc} 8\pi p_{r} & =-\frac{1}{r^{2}}+(\frac{e^{-\lambda }\nu^{\prime }}{r}+\frac{e^{-\lambda }}{r^{2}}), \end{array}$$ (5)

$$\begin{array}{cc} 8\pi p_{t} & =\frac{1}{4}(\nu^{\prime 2}+2\nu^{\prime \prime }-\nu^{\prime }\lambda^{\prime }+\frac{2\nu^{\prime }}{r}-\frac{2\lambda^{\prime }}{r})e^{-\lambda }. \end{array}$$ (6)

Throughout the analysis, (′) denotes differentiation with respect to the radial coordinate *r*. Upon simplifying the resulting field Eqs. (4)–(6), the following relations are obtained, consistent with the formulation presented in [41].


$$\begin{array}{c} e^{\lambda }=(1-\frac{2M(r)}{r}{)}^{-1}, \end{array}$$ (7)

$$\begin{array}{c} 8\pi p_{r}=(1-\frac{2M(r)}{r})\frac{\nu^{\prime }}{r}-\frac{2M(r)}{r^{3}}, \end{array}$$ (8)

$$\begin{array}{c} (p_{r}+\rho )\nu^{\prime }+2p_{r}^{\prime }=-\frac{4}{r}\sqrt{3}S. \end{array}$$ (9)

The mass function is written as


$$\begin{array}{c} M(r)=4\pi \int_{0}^{r}r^{2}\rho dr. \end{array}$$ (10)

A single polytropic EoS is a phenomenological representation and cannot reproduce the complete microphysics of cold, β-equilibrated neutron-star matter over the entire density range. Realistic relativistic mean-field (RMF) equations of state determine the pressure from an interacting nuclear Lagrangian and incorporate nuclear saturation properties, symmetry-energy effects, density-dependent scalar and vector interactions, and possible changes in the particle composition [42–44]. Their effective pressure $p_{r}$ response therefore varies with density ρ, described through the polytropic EoS [45] as


$$\begin{array}{c} p_{r}=\alpha \rho^{\gamma }. \end{array}$$ (11)

Here, $\gamma =1+\frac{1}{n}$ and *n* represent a polytropic index. The $p_{r}$ response, which is also the squared radial sound speed in geometrized units, is given by


$$\begin{array}{c} v_{r}^{2}=\frac{dp_{r}}{d\rho }=\alpha \gamma \rho^{\gamma -1}=\gamma \frac{p_{r}}{\rho }. \end{array}$$

The behavior of EoS is determined jointly by γ, the polytropic coefficient α, and the density interval under consideration. A comparison based only on the exponent is therefore qualitative and does not provide a one-to-one correspondence with a particular RMF parametrization. Furthermore, the physically admissible parameter values must satisfy the causal condition


$$\begin{array}{c} 0\leq v_{r}^{2}\leq 1 \end{array}$$

throughout the stellar interior. This condition has been verified for all parameter sets used in the numerical analysis. The representative values considered in the present work are


$$\begin{array}{c} n=\frac{1}{2},\;1,\;\frac{3}{2},\;\gamma =3,\;2,\;\frac{5}{3}, \end{array}$$

respectively. For consistently normalized and physically admissible values of α, these choices sample progressively weaker local pressure responses as *n* increases. The case *n* = 1/2 provides the strongest pressure response among the selected models, while *n* = 1 represents an intermediate benchmark frequently employed in relativistic stellar modeling. The case *n* = 3/2 gives the weakest response among the three choices and is historically associated with a non relativistic degenerate Fermi gas in Newtonian polytropic theory. This historical association is used only to motivate the selected exponent and should not be interpreted as implying that the core of PSR J0952–0607 behaves as an ideal non relativistic Fermi gas. For clarity, the qualitative roles of the selected indices are summarized in Table 1. For comparison with microscopic equations of state, it is useful to introduce the logarithmic pressure-response index


$$\begin{array}{c} \gamma_{log}(\rho )=\frac{d\ln p}{d\ln\rho }. \end{array}$$

**Table 1:** Selected polytropic indices and their qualitative pressure responses.

*n*	γ=1+1/n	Qualitative pressure response
$\frac{1}{2}$	3	Strongest among the selected cases
1	2	Intermediate
$\frac{3}{2}$	$\frac{5}{3}$	Weakest among the selected cases

For the present polytropic model,


$$\begin{array}{c} \gamma_{log}=\Gamma , \end{array}$$

and is therefore constant. In contrast, for an RMF EoS, $\gamma_{log}$ generally varies with density because of the competing effects of scalar attraction, vector repulsion, symmetry-energy contributions, and changes in particle composition [42–44,46]. The quantity $\gamma_{log}$ should not be confused with the relativistic adiabatic index used in the stability analysis,


$$\begin{array}{c} \gamma_{r}=\frac{\rho +p_{r}}{p_{r}}\frac{dp_{r}}{d\rho }, \end{array}$$

which is evaluated separately in next section. Polytropic representations nevertheless remain useful as controlled approximations in neutron-star modeling. Read *et al.* showed that realistic high-density equations of state can be represented accurately by piecewise-polytropic forms over different density intervals [47]. Subsequent parametrized and generalized piecewise-polytropic formulations have been developed to reproduce neutron-star masses, radii, tidal deformabilities, oscillation frequencies, continuous sound-speed behavior, and thermodynamic properties [48,49]. Accordingly, the present constant-γ cases should be regarded as local phenomenological surrogates that sample comparatively stronger, intermediate, and weaker pressure responses. They do not reproduce the complete nuclear saturation properties, composition dependence, phase thresholds, or density-dependent sound speed of any specific RMF EoS.

The present study is not intended as a microscopic reconstruction of PSR J0952–0607. Rather, it is a controlled phenomenological investigation of how the generalized Tolman–Kuchowicz geometry responds to different effective matter compressibilities. A quantitatively realistic description of PSR J0952–0607 would require either a piecewise-polytropic parametrization calibrated to a microscopic nuclear EoS [47–49], or the direct implementation of a causal RMF EoS satisfying both nuclear and astrophysical constraints [42–44,46].

## 3. Solutions of the EFEs in the GTK metric

By inserting the polytropic EoS given in Eq. (11) together with the GTK metric functions in Eq. (2) into the EFEs (4–6), the following set of expressions is obtained as


$$\begin{array}{c} \rho =\frac{1-(1+ar^{2}+br^{4}{)}^{-m}}{r^{2}}+2m(a+2br^{2})(1+ar^{2}+br^{4}{)}^{-1-m}, \end{array}$$ (12)

$$\begin{array}{c} p_{r}=[\frac{1-(1+ar^{2}+br^{4}{)}^{-m}}{r^{2}}+2m(a+2br^{2})(1+ar^{2}+br^{4}{)}^{-1-m}{]}^{1+1/n}\alpha , \end{array}$$ (13)

where $n=1,\frac{1}{2},\;\frac{3}{2}$ [50]. Differentiating Eq. (13) with respect to *r* leads to


$$\begin{array}{cc} \frac{dp_{r}}{dr} & =\alpha \left(1+\frac{1}{n}\right){\left(2myx^{-1-m}+\frac{1}{r^{2}}+-\frac{x^{-m}}{r^{2}}\right)}^{\frac{1}{n}}\\ & \left(-\frac{2}{r^{3}}-\frac{2m(1+m)yz}{x^{2+m}}+8bmrx^{-1-m}+\frac{mzx^{-(1+m)}}{r^{2}}+\frac{2x^{-m}}{r^{3}}\right) \end{array}$$ (14)

Here, $x=1+ar^{2}+br^{4},$ *y* = *a* + 2*br*^2^, and *z* = 2*ar* + 4*br*^3^. By using Eqs. (11)–(13) with (9), we get


$$\begin{array}{cc} \sqrt{3}\;S^{*} & =-\frac{1}{2}r[2q^{1/n}\alpha \left(1+\frac{1}{n}\right)(-\frac{2}{r^{3}}+2(-1-m)myzx^{-(2+m)}+8bmrx^{-(1+m)}\\ & +\frac{mzx^{-(1+m)}}{r^{2}}+\frac{2}{x^{m}r^{3}})+\frac{1}{2\left(3-8\pi r\left(\frac{1}{r^{2}}+2nyx^{-1-n}-\frac{x^{-n}}{r^{2}}\right)\right)}\\ & \times \left(\frac{1}{r^{2}}+\frac{2my}{x^{1+m}}-\frac{1}{x^{m}r^{2}}+q^{1+\frac{1}{n}}\alpha \right)\left(8\pi r\left(\frac{1}{r^{2}}+\frac{2ny}{x^{1+n}}-\frac{1}{x^{n}r^{2}}\right)+24\pi q^{1+\frac{1}{n}}\alpha \right)] \end{array}$$ (15)

The anisotropy vanishes in the center *r* = 0. We can derive $p_{t}$ by substituting the GTK metric potentials given in Eq. (2) into the EFEs. The temporal field equation determines ρ, while $p_{r}$ is obtained by imposing the polytropic EoS. After fixing ρ and $p_{r}$ the $p_{t}$ is determined by the angular component of the Einstein Eq. (6). Equivalently, using the conservation equation


$$\begin{array}{c} \frac{dp_{r}}{dr}+\frac{\nu^{\prime }}{2}(\rho +p_{r})-\frac{2}{r}(p_{t}-p_{r})=0, \end{array}$$ (16)

one obtains


$$\begin{array}{c} p_{t}=p_{r}+\frac{r}{2}\frac{dp_{r}}{dr}+\frac{r\nu^{\prime }}{4}(\rho +p_{r}). \end{array}$$

The anisotropy $\Delta =p_{t}-p_{r}$ is therefore a derived quantity and not an independently imposed matter function. The relation $p_{t}=p_{r}-\sqrt{3}S^{*}$ is given as


$$\begin{array}{cc} p_{t} & =q^{1+1/n}\alpha -\frac{r}{2}[2q^{1/n}\alpha \left(\frac{n+1}{n}\right)(-\frac{2}{r^{3}}+-2(1+m)myzx^{-(2+m)}\\ & +\frac{8bmr}{x^{1+m}}+\frac{mz}{x^{\left(1+m\right)}r^{2}}+\frac{2x^{-m}}{r^{3}})+\frac{1}{2\left(3-8\pi r\left(\frac{1}{r^{2}}+\frac{2ny}{x^{1+n}}-\frac{x^{-n}}{r^{2}}\right)\right)}\\ & \times \left(\frac{1}{r^{2}}+\frac{2my}{x^{1+m}}-\frac{1}{x^{m}r^{2}}+q^{1+\frac{1}{n}}\alpha \right)(8\pi r\left(\frac{2ny}{x^{-(1+n)}}+\frac{1}{r^{2}}-\frac{1}{x^{n}r^{2}}\right)\\ & +24\alpha \pi q^{1+\frac{1}{n}})], \end{array}$$ (17)

where, q=$\left(\frac{1}{r^{2}}+2myx^{-1-m}-\frac{x^{-m}}{r^{2}}\right).$ We explicitly state in Section 2 that the GTK metric potentials are assumed a priori. Then clarify in Section 3 that the polytropic EoS serves only as a parametric relation used to express $p_{r}$ and ρ in closed form and to constrain the constants through surface boundary conditions $p_{r}(R)=0$.

## 4. Matching conditions for model parameters

To determine the central density ($\rho_{c}$), Eq. (12) is expanded in a Taylor series about *r* = 0, yielding


$$\begin{array}{c} \rho_{c}=2am. \end{array}$$ (18)

Equations (13) and (17) satisfy the condition


$$\begin{array}{c} p_{r}(0)=p_{t}(0). \end{array}$$ (19)

The parameters *a*, *b*, *A*, and *B* are determined by applying the suitable boundary conditions. These conditions are enforced at the boundary surface of star at *r* = *R*.

At this radius, the metric describing the star’s interior (the GTK solution) must transition smoothly into the exterior Schwarzschild geometry [51]. This matching guarantees that the spacetime remains physically continuous across the boundary.


$$\begin{array}{c} ds^{2}=(1-\frac{2M}{r})\;dt^{2}-\frac{1}{\left(1-\frac{2M}{r}\right)}\;dr^{2}-r^{2}\left(d\theta^{2}+\sin^{2}\theta \;d\phi^{2}\right). \end{array}$$ (20)

Here, *M* represents the total gravitational mass of the star. The smooth junction conditions at the stellar radius *r* = *R* guarantee the continuity of the metric functions at the boundary. Specifically, the interior radial and temporal metric components must match those of the Schwarzschild exterior, thereby guaranteeing a regular junction between interior and exterior spacetime geometry.


$$\begin{array}{c} 1-\frac{2M}{R}=A^{2}e^{BR^{2}}, \end{array}$$ (21)

$$\begin{array}{c} (1-\frac{2M}{R}{)}^{-1}=(1+aR^{2}+bR^{4}{)}^{m}, \end{array}$$ (22)

$$\begin{array}{c} \frac{\partial (g_{tt})}{\partial r}=\frac{M}{R}=BR^{2}e^{BR^{2}}. \end{array}$$ (23)

At the star’s surface, the $p_{r}$ becomes zero, leading to


$$\begin{array}{c} p_{r}(R)=0. \end{array}$$ (24)

Eqs. (21)–(23) can now be used to evaluate the constants *a*, *A*, *b*, and *B* such as


$$\begin{array}{c} A=\left(e^{-\frac{BR^{2}}{2}}\right){\left(-\frac{2M}{R}+1\right)}^{1/2}, \end{array}$$ (25)

$$\begin{array}{c} B=\frac{M}{R^{3}\left(-\frac{2M}{R}+1\right)}, \end{array}$$ (26)

$$\begin{array}{c} a=\frac{U^{-(1+m)}}{2m(1-U^{-m})}+\frac{2(U-1)}{r^{2}}, \end{array}$$ (27)

$$\begin{array}{c} b=\frac{-1-aR^{2}+U}{R^{4}}. \end{array}$$ (28)

Here, $U={\left(1-\frac{2M}{R}\right)}^{-\frac{1}{m}}$. The constants *a*, *A*, *b*, and *B* can be calculated using Eqs. (25)–(28). This is accomplished by choosing suitable values for the parameters α, *m*, and *n*.

## 5. Physical analysis of the model

In this section, we investigate the physical characteristics of compact objects within a static and spherically symmetric spacetime. To evaluate the viability of the model, the compact object PSR J0952-0607 is adopted as a test case, with an observed mass and radius as $2.35\pm 0.17\;M_{⊙}$ and $13.7_{-1.5}^{+2.6}\;km$, respectively [10]. Consistent with the polytropic EoS parameter *n* and GTK metric exponent *m*, the analysis is carried out for *n* = 1, $\frac{1}{2}$, and $\frac{3}{2}$ with 1≤m≤3. The physical quantities including ρ, $p_{r}$, and $p_{t}$, we present their radial profiles graphically. Additional plots illustrate Δ, the causality conditions and the energy conditions. In particular, the GTK exponent *m* enters the radial metric potential given in Eq. (2) and therefore regulates the curvature and the effective strength of the interior gravitational field. This dependence is transferred directly to ρ expressed in Eq. (12). This equation gives the central value $\rho_{c}=2am$. So, for fixed admissible values of the remaining constants, a larger *m* enhances the central concentration of matter and increases the magnitude of the outward density gradient. The corresponding stronger gravitational attraction requires a larger pressure-gradient contribution and, in the anisotropic case, an additional transverse stress contribution to maintain hydrostatic equilibrium. The parameter *n*, on the other hand, determines the effective response of the matter sector through polytropic EOS. It therefore controls the sensitivity of the radial pressure to changes in density and consequently affects the pressure gradients, radial sound speed and adiabatic response of the fluid. The tangential pressure is not imposed by a separate EoS, but is generated by the remaining Einstein equation together with the conservation law. Thus, the anisotropy measures the difference between the transverse stress required by the GTK curvature and the radial stress supplied by the polytropic matter relation. The surfaces presented below should therefore be interpreted as manifestations of this geometry-matter coupling: *m* primarily controls the gravitational concentration and radial curvature, whereas *n* controls the stiffness and compressional response of the matter distribution. The monotonic decrease of ρ, $p_{r}$ and $p_{t}$ is physically associated with the gradual weakening of the gravitational field from the center toward the stellar surface. Near *r* = 0, the regularity of the GTK potential gives finite central values and vanishing first derivatives, whereas away from the center the factor $(1+ar^{2}+br^{4}{)}^{-m}$ suppresses the geometric contribution to the density. A larger value of *m* amplifies this suppression and produces the steeper density gradients. Through the polytropic relation, the same density decrease is transmitted to the radial pressure. However, because the exponent 1 + 1/*n* depends on *n*, different polytropic indices convert the same geometrically generated density profile into different pressure responses. The transverse pressure additionally contains curvature and anisotropic contributions, and therefore does not follow the radial pressure identically. Their difference remains zero at the center, as required by spherical symmetry, but grows outward as the radial and transverse components of the Einstein equations respond differently to the nonuniform matter distribution.

### 5.1 Central energy density and pressure profile

The central energy density ($\rho_{c}$) can be determined from Eq. (12)


$$\begin{array}{c} \rho_{c}=2am. \end{array}$$ (29)

Using Eq. (29) in Eq. (11), central pressure $\left(p_{rc}\right)$ is evaluated as


$$\begin{array}{c} p_{rc}=(2am{)}^{1+\frac{1}{n}}\;\alpha . \end{array}$$ (30)

Fig 1 shows that the energy density ρ is maximum at the stellar center and decreases monotonically with increasing radial coordinate *r*, remaining positive throughout the interior. Higher values of the GTK exponent *m* correspond to larger central densities, indicating that *m* significantly influences the compactness and internal matter distribution of PSR J0952–0607.

**Fig 1:**
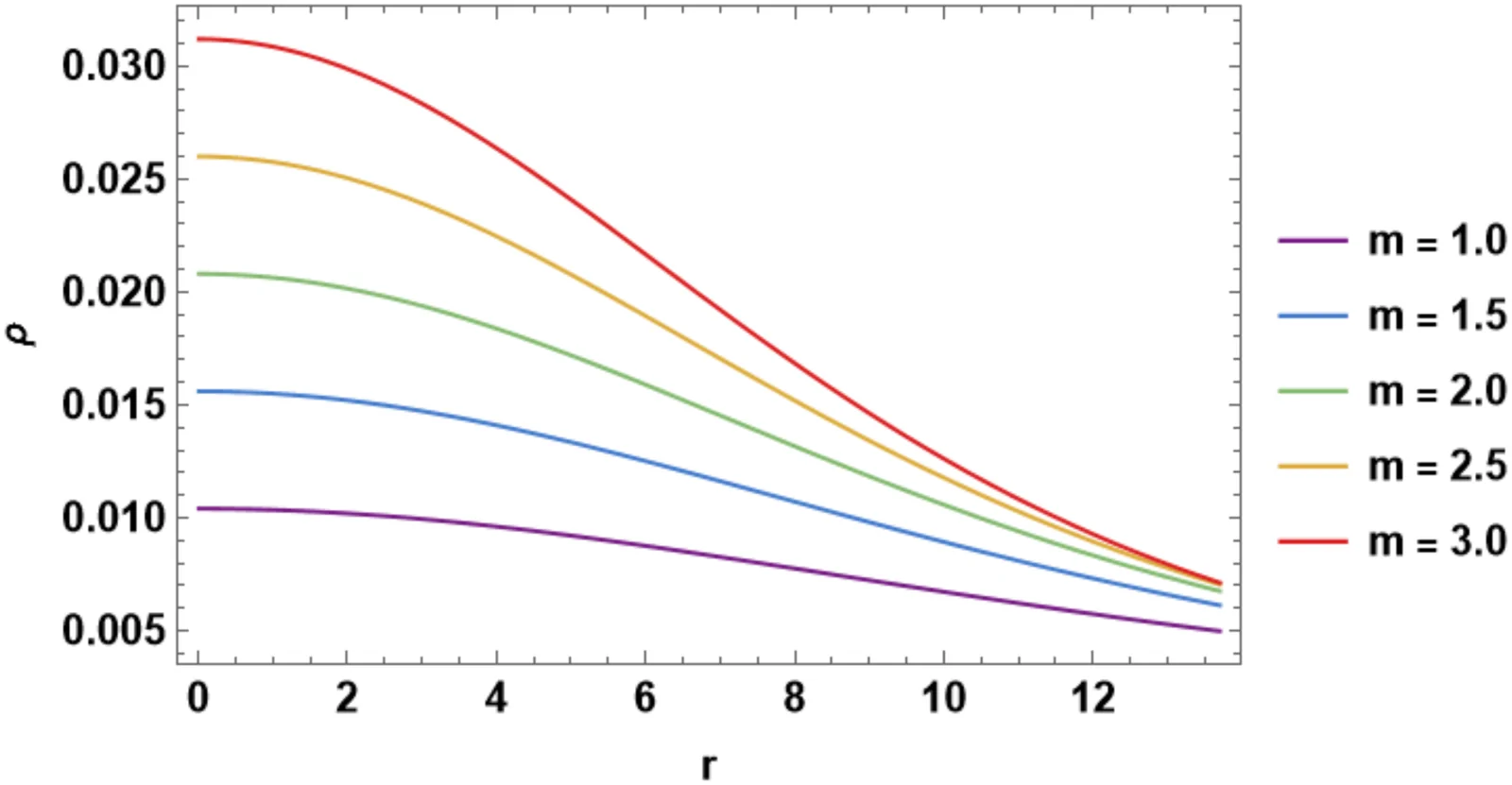
Radial profile of energy density $\rho \;(km^{-2})$ of PSR J0952–0607 for different values of *m.*

Fig 2 represents how the density gradient $\left(\frac{d\rho }{dr}\right)$ varies with radius *r* for different GTK exponents *m* for PSR J0952-0607. The density gradient is zero at the stellar center (*r* = 0), which makes it regular. Throughout the interior, $\left(\frac{d\rho }{dr}\right)$ is negative, which means that the energy density decreases monotonically outward to the surface, which is also a necessary physical condition. Increasing the value of *m* enhances the magnitude of the negative gradient, reflecting a steeper radial decrease in density while preserving smooth behavior across the stellar configuration.

**Fig 2:**
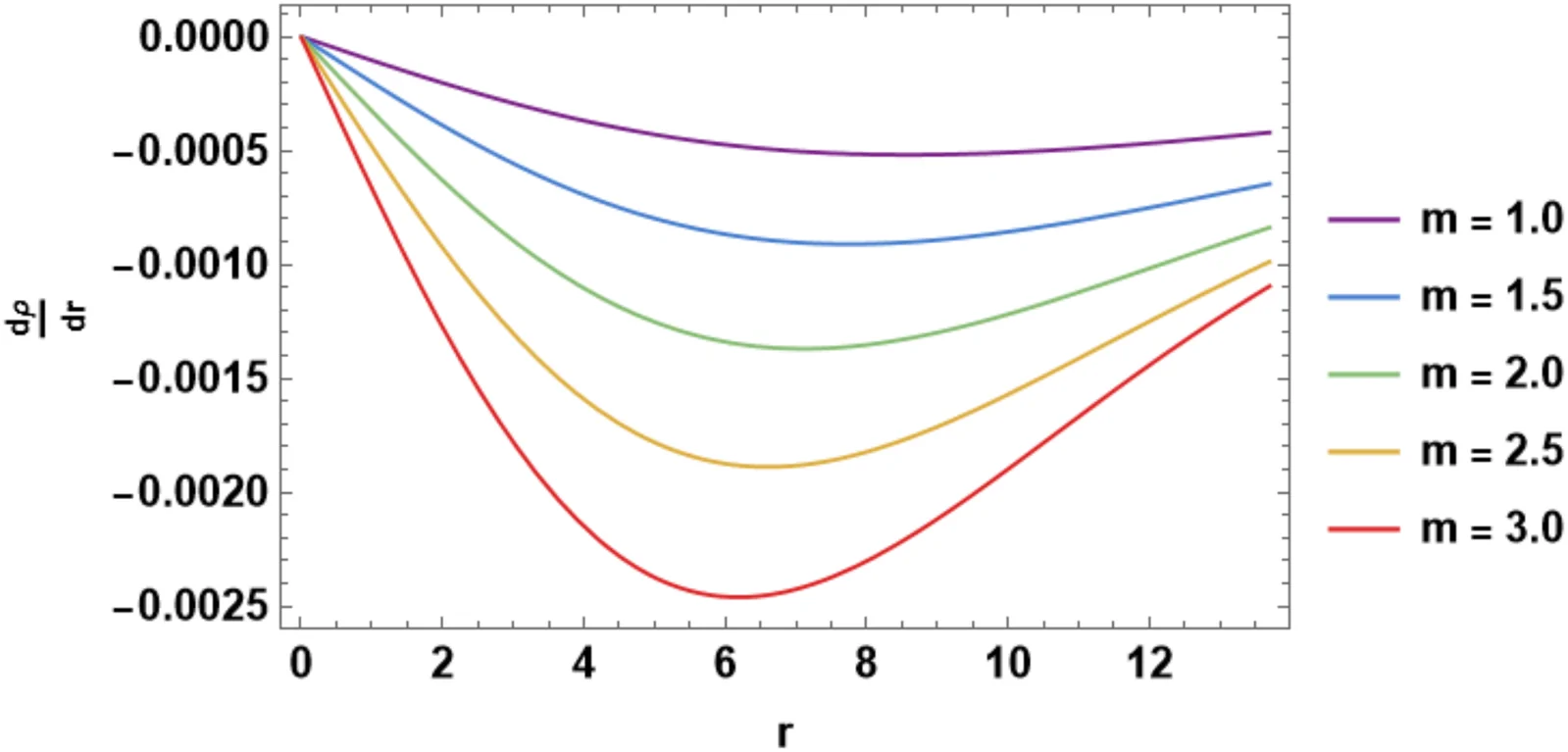
Density gradient fulfilling regularity condition $d\rho /dr<0\;(km^{-3})$, for different values of *m.*

Fig 3 show the behavior of $p_{r}$. This pressure profile exhibit a decreasing behavior of the surfaces as *n* and *m* increase. The surfaces of $p_{r}$ in Fig 3 vanish on the star boundary.

**Fig 3:**
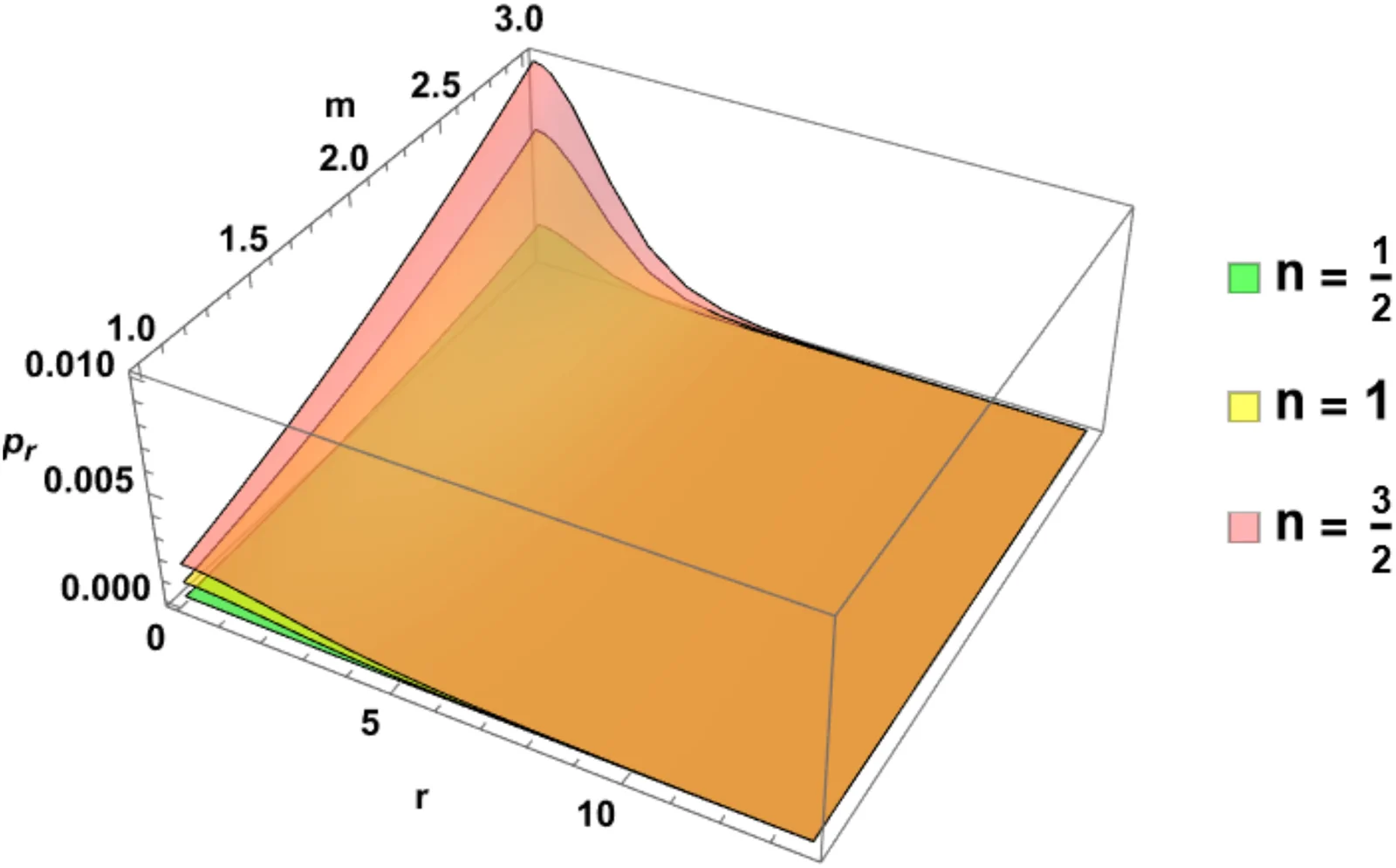
Variation of the radial pressure $p_{r}\;(km^{-2})$ for PSR J0952–0607, decreasing monotonically to zero at the surface for different polytropic indices *n.*

Fig 4 present the behavior of $p_{t}$ with radial coordinate *r*. Fig 4 surfaces illustrate the decreasing behavior of $p_{t}$ with increasing values of *m* and *n*. The ρ, $p_{r}$, and $p_{t}$ remain finite, continuous, and well behaved within the stellar interior. These quantities attain their maximum values at the center and decrease smoothly toward the boundary.

**Fig 4:**
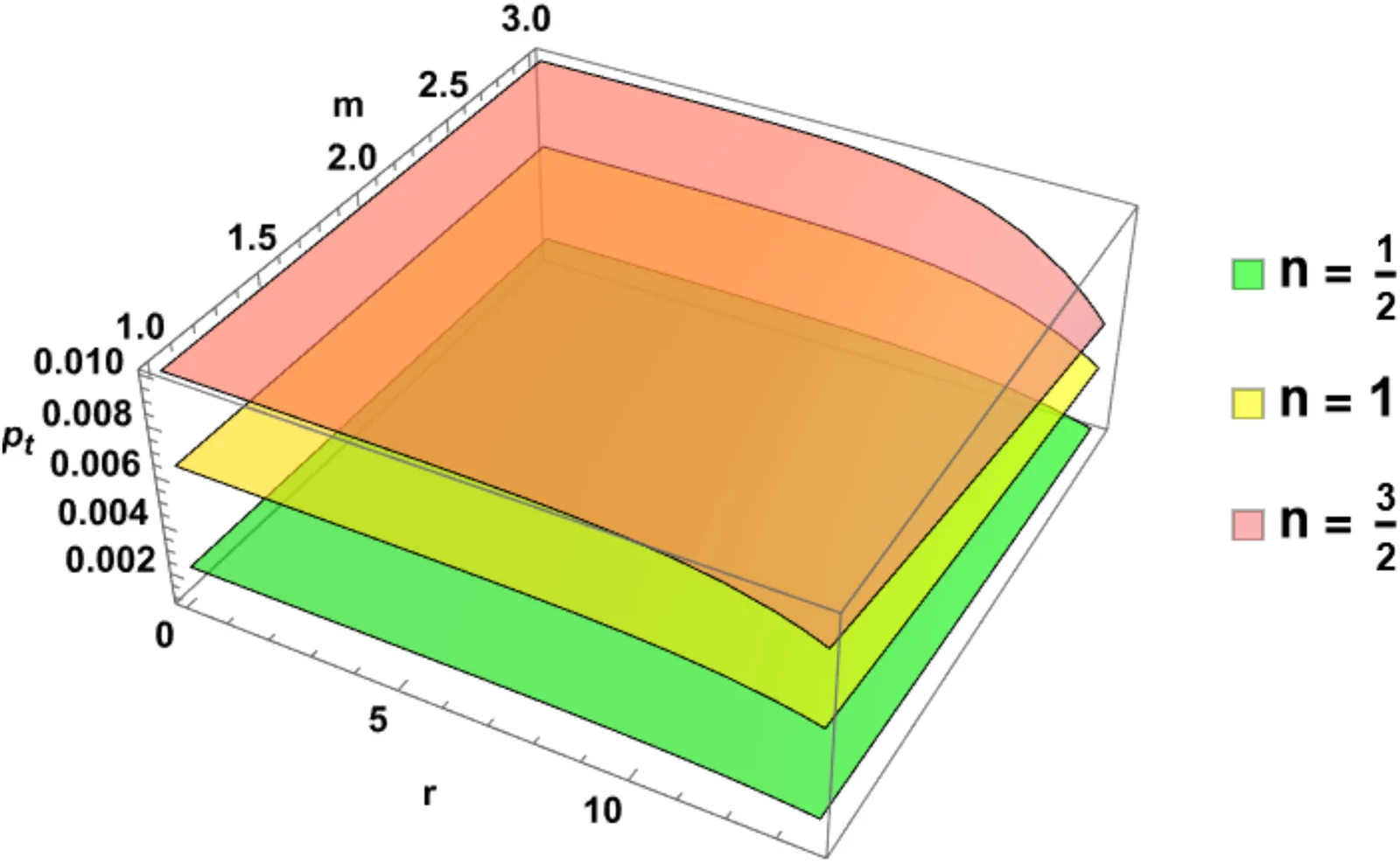
Radial variation of the tangential pressure $p_{t}\;(km^{-2})$ for PSR J0952–0607, decreasing smoothly to zero at the surface for different polytropic indices *n.*

Fig 5 shows the radial behavior of Δ for different values of the polytropic index *n*. For $n=\frac{1}{2},\;1,\;\frac{3}{2}$, each surface traced the behavior of Δ from center to the stellar boundary.

**Fig 5:**
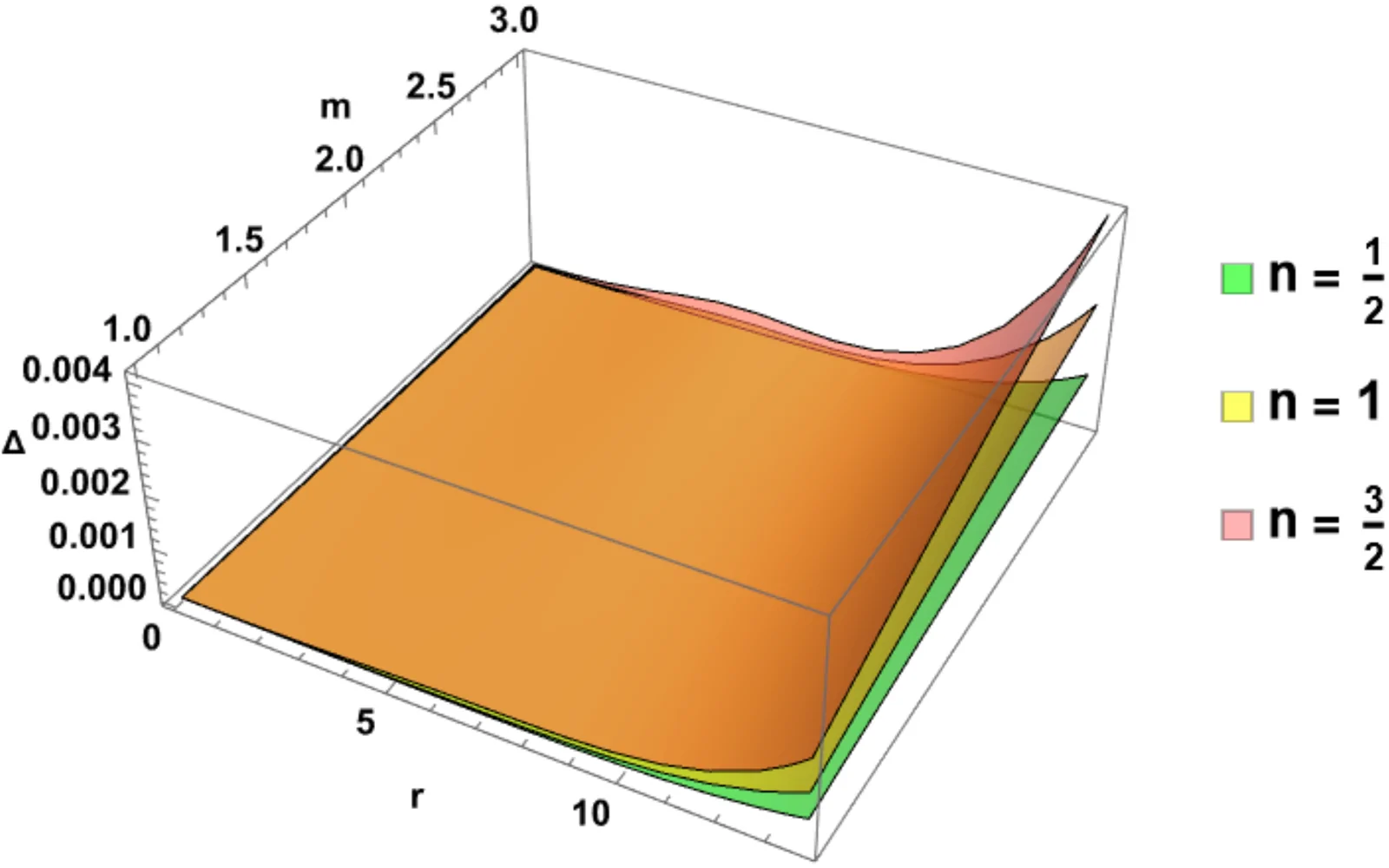
Radial profile of anisotropy factor $\Delta \;(km^{-2})$ for different values of *n.*

At *r* = 0, all profiles satisfy Δ=0, indicating isotropic pressure, after which the anisotropy develops gradually with increasing radius. The $p_{r}$ is prescribed through the polytropic EoS whereas the $p_{t}$ is obtained from the angular component of EFEs after introducing the GTK metric potentials. Consequently, anisotropy is a derived effective anisotropic stress compatible with the selected spacetime geometry, radial EoS, field equations, and boundary conditions. It is not introduced as an independent ansatz and is not derived from a unique microscopic Hamiltonian or from an explicitly magnetized, superfluid, superconducting, or elastic matter Lagrangian. Nevertheless, unequal principal stresses are physically plausible in neutron-star matter. Ruderman [16] noted that matter at supranuclear densities may develop anisotropic stresses because of relativistic nuclear interactions and the possible formation of solid, superfluid, or magnetized phases. The consequences of unequal radial and tangential stresses for relativistic hydrostatic equilibrium were subsequently formulated by Bowers and Liang [52]. A strong internal magnetic field provides a particularly clear microscopic source of pressure anisotropy because it introduces a preferred spatial direction. The corresponding matter and electromagnetic stresses generally separate into components parallel and perpendicular to the magnetic field, yielding $p_{r}\text{≠}p_{t}$ [53,54]. Direction-dependent stresses may also arise from neutron superfluidity and proton superconductivity through quantized vortices, magnetic-flux tubes, anisotropic pairing, and entrainment effects [55,56]. Furthermore, transitions to hyperonic matter, meson condensates, deconfined quark matter, mixed phases, or crystalline phases may produce effective anisotropic stresses in the high-density stellar interior [57,58]. In the present model, these mechanisms should be regarded as possible physical motivations rather than as a unique microscopic derivation of the calculated anisotropy, Δ(r). The anisotropy therefore represents a phenomenological macroscopic description of unresolved direction-dependent stresses. We have now stated this limitation explicitly in the revised manuscript and clarified that a definitive identification of the microscopic origin would require an extended model containing, for example, an electromagnetic field, a multifluid superfluid description, an elastic strain tensor, or a phase-transition order parameter. In addition, the calculated profile satisfies


$$\begin{array}{c} \Delta (0)=0, \end{array}$$

as required by regularity and spherical symmetry. The numerical analysis further shows that Δ(r) remains positive and increases outward throughout the stellar interior. Consequently, $p_{t}>p_{r}$, and the anisotropic contribution acts in the outward direction. Its mechanical influence is described through the anisotropic term in the TOV equilibrium equation,


$$\begin{array}{c} F_{a}=\frac{2\Delta }{r}, \end{array}$$

which quantifies how the derived transverse stress contributes to the internal hydrostatic balance [16,53–55,57,58].

Fig 6 demonstrate how the radial pressure gradient $\frac{dp_{r}}{dr}$ varies under different polytropic indices $n=1,\;\frac{1}{2},\;\frac{3}{2}$ and GTK exponent *m*.

**Fig 6:**
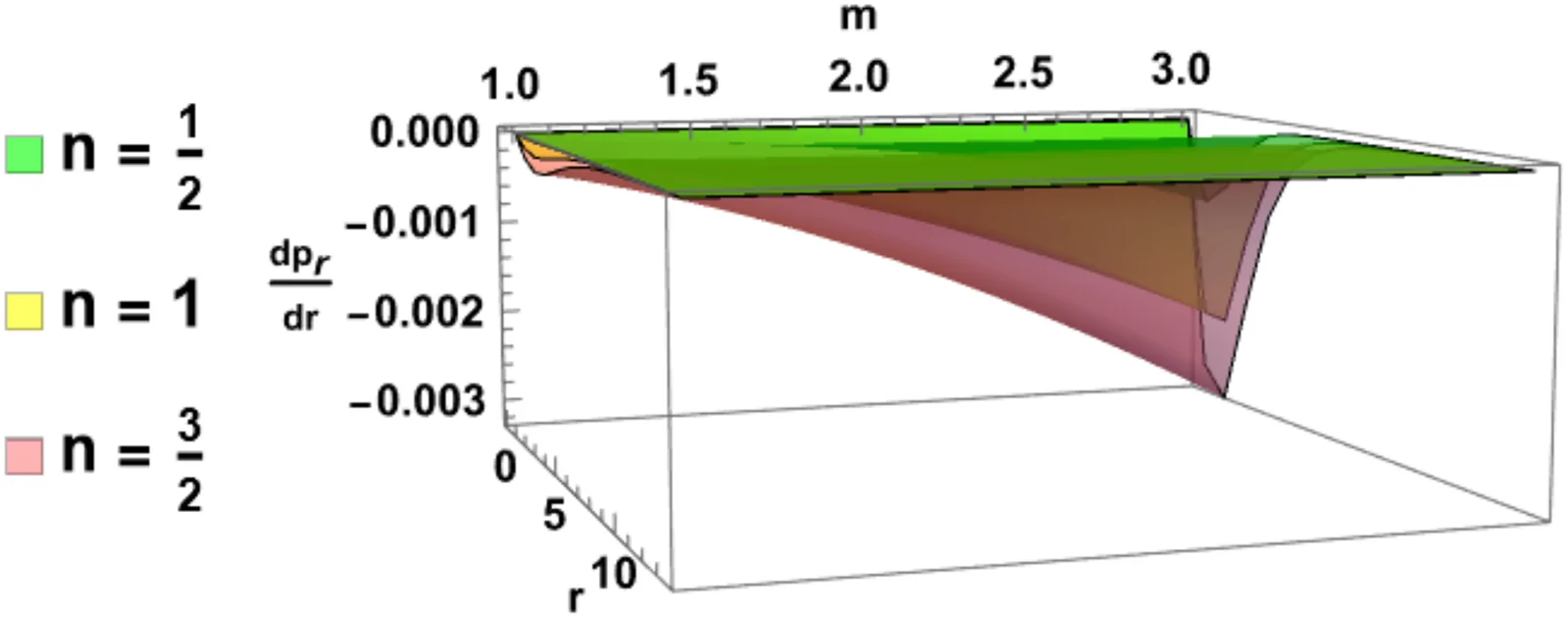
Radial pressure gradient fulfilling regularity condition $dp_{r}/dr<0\;(km^{-3})$, for different polytropic indices *n.*

Fig 7 shows the gradient of tangential pressure $\frac{dp_{t}}{dr}$ with radius *r* for the compact star PSR J0952-0607 under different polytropic indices *n* and GTK metric exponent *m*. The three dimensional surface plot demonstrates that $\frac{dp_{t}}{dr}$ decreases with radius, more dramatically for higher polytropic indices. As in $p_{t}$ for *n* = 3/2 causes a stronger gradient, indicating a greater pressure anisotropy deeper inside the star. The figures depend on several parameters such as $M=2.35\;M_{⊙}$, *R* = 13.7 *km*, *A* = 0.5434, *B* = 0.002735, *a* = 0.016482, *b* = 0.05, α=0.007 with the GTK exponent range 1≤m≤3.

**Fig 7:**
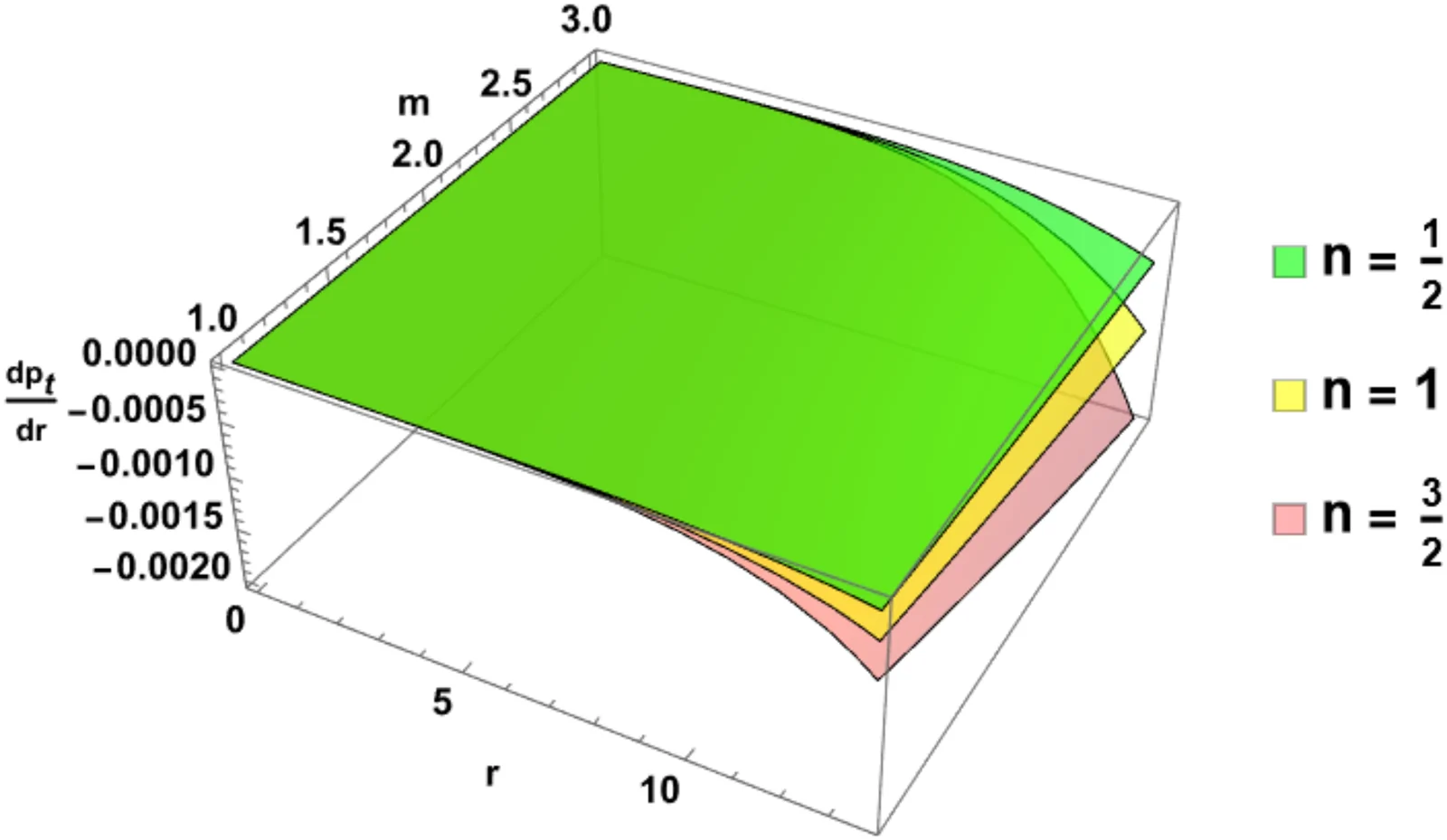
Tangential pressure gradient fulfilling regularity condition $dp_{t}/dr<0\;(km^{-3})$, for different polytropic indices *n.*

### 5.2 Herrera cracking technique and causality conditions

To further ensure the stability of a physically viable compact stellar object, we also performed a graphical analysis based on numerical values assigned to several model parameters. This assessment is carried out by verifying the causality condition, which serves as a key indicator of physical stability. In particular, we examine the nature of the sound speed in stellar interior. To meet the causality condition, $v_{r}^{2}$ and $v_{t}^{2}$ must satisfy the inequalities $0<v_{r}^{2}<1$ and $0<v_{t}^{2}<1$ at all points in the stellar interior [59,60].

The mathematical expressions are given as follows.


$$\begin{array}{c} v_{r}^{2}=\frac{dp_{r}}{d\rho } \end{array}$$ (31)

$$\begin{array}{c} v_{t}^{2}=\frac{dp_{t}}{d\rho } \end{array}$$ (32)

The surface in Fig 8 illustrate the behavior of $v_{r}^{2}$ for PSR J0952-0607. The surface plot confirm that $v_{r}^{2}$ consistently lie within the physically acceptable range of sound speed.

**Fig 8:**
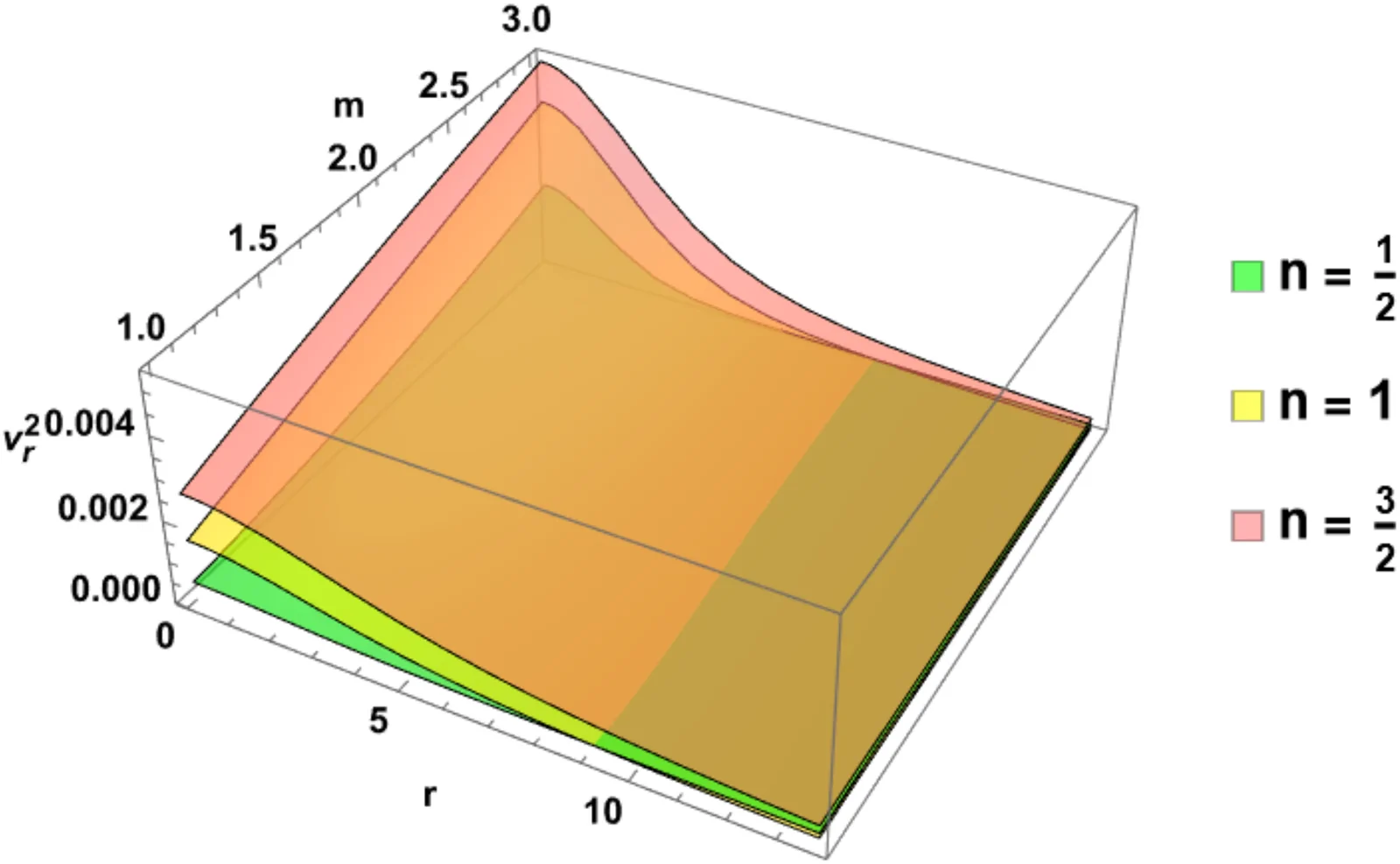
Variation of the radial sound speed which remains within the causal bound $0<v_{r}^{2}<1$ for different polytropic indices *n.*

The surface in Fig 9 illustrate the behavior of $v_{t}^{2}$ for PSR J0952-0607. The surface plot confirm that $v_{t}^{2}$ consistently lie within the physically acceptable range of sound speed.

**Fig 9:**
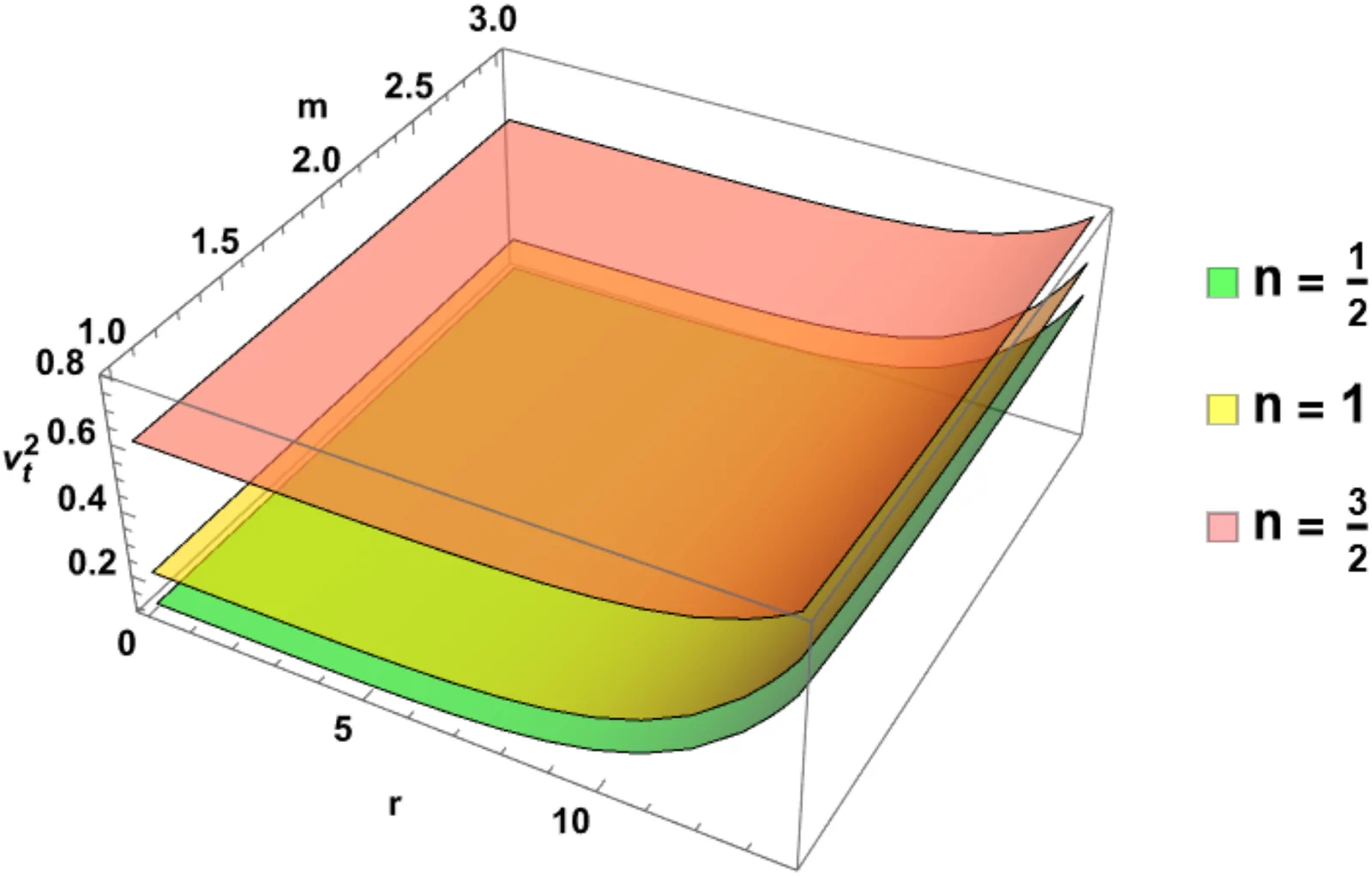
Radial variation of the tangential sound speed $v_{t}^{2}$ for PSR J0952–0607, which remains within the causal bound $0<v_{t}^{2}<1$ for different polytropic indices *n.*

Furthermore, the stability analysis integrates Herrera’s cracking technique with Abreu’s criterion, which is expressed mathematically as


$$\begin{array}{c} 0<|v_{t}^{2}-v_{r}^{2}|<1. \end{array}$$

For different polytropic indices *n*, Fig 10 demonstrates that $v_{t}^{2}-v_{r}^{2}$ surfaces are positive throughout the star’s interior, suggesting that $v_{t}^{2}$ is greater than $v_{r}^{2}$. Abreu’s stability criterion is satisfied by this behavior, indicating that PSR J0952-0607 has a stable configuration.

**Fig 10:**
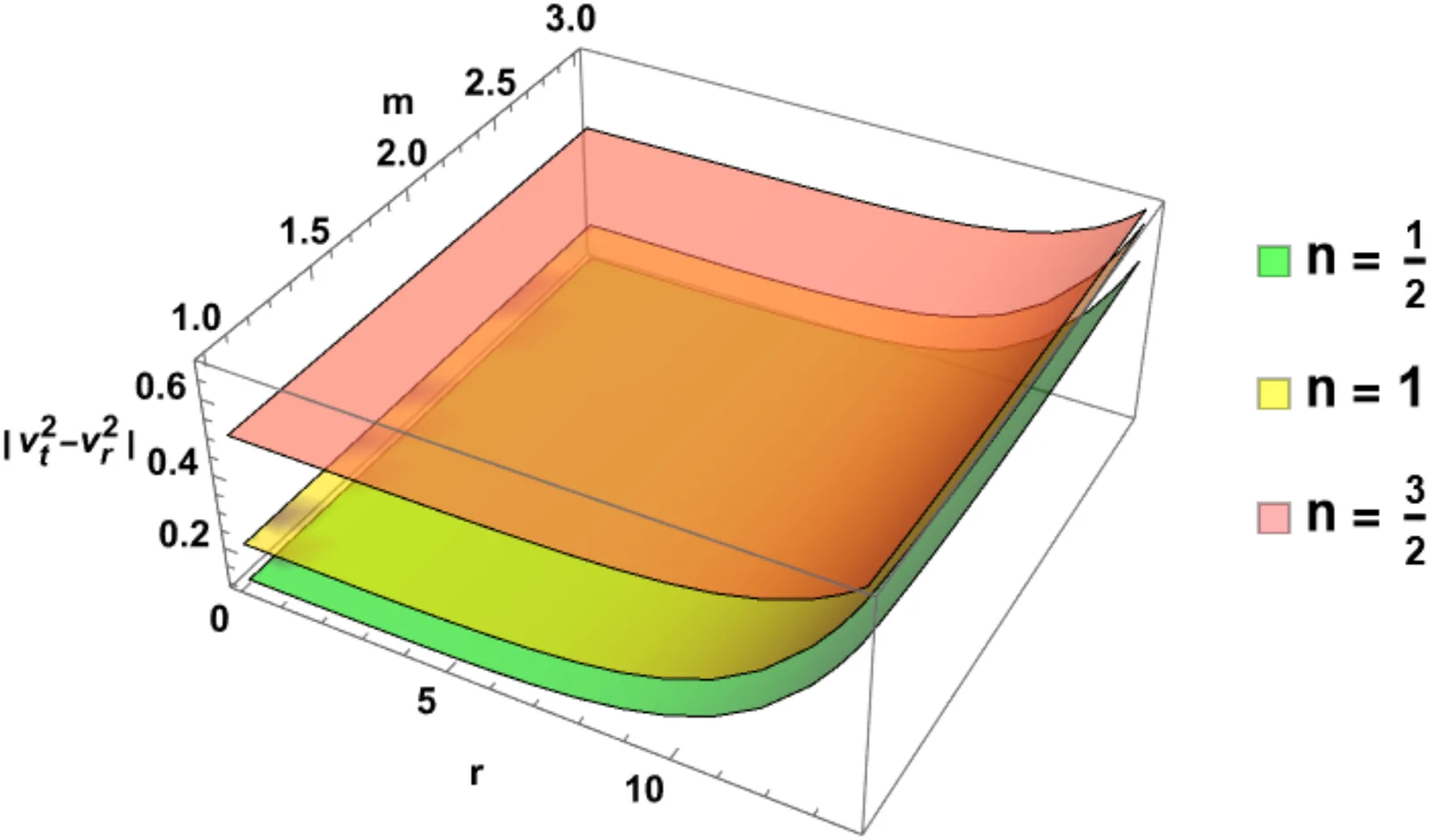
Radial variation of $\left|v_{t}^{2}-v_{r}^{2}\right|$ for PSR J0952–0607, confirming stability under Herrera’s cracking criterion.

### 5.3 Adiabatic index

The adiabatic index tests the stability of compact stars both in relativistic and classical theories. Since the ratio of specific heats, Γ is a crucial parameter necessary to describe the EoS in various density regimes. The first to propose the concept of using the quantity of Γ as a measure of stability to infinitesimal radial perturbations was Chandrasekhar [61]. It has since been widely studied in many astrophysical studies [62–65]. These analyzes indicate that a stellar configuration is dynamically stable only when $\Gamma >\frac{4}{3}$ is in the interior of a star. The $\Gamma_{r}$ and $\Gamma_{t}$ can be expressed as


$$\begin{array}{c} \Gamma_{r}=(\frac{\rho +p_{r}}{p_{r}})v_{r}^{2}, \end{array}$$ (33)

$$\begin{array}{c} \Gamma_{t}=(\frac{\rho +p_{t}}{p_{t}})v_{t}^{2}. \end{array}$$ (34)

Figs 11 and 12 illustrate the behavior of the radial $\Gamma_{r}$ and transverse $\Gamma_{t}$ adiabatic indices, respectively, showing that both remain above the critical threshold of $\frac{4}{3}$ throughout the interior of PSR J0952-0607. This confirms that the proposed model satisfies the criterion for dynamical stability.

**Fig 11:**
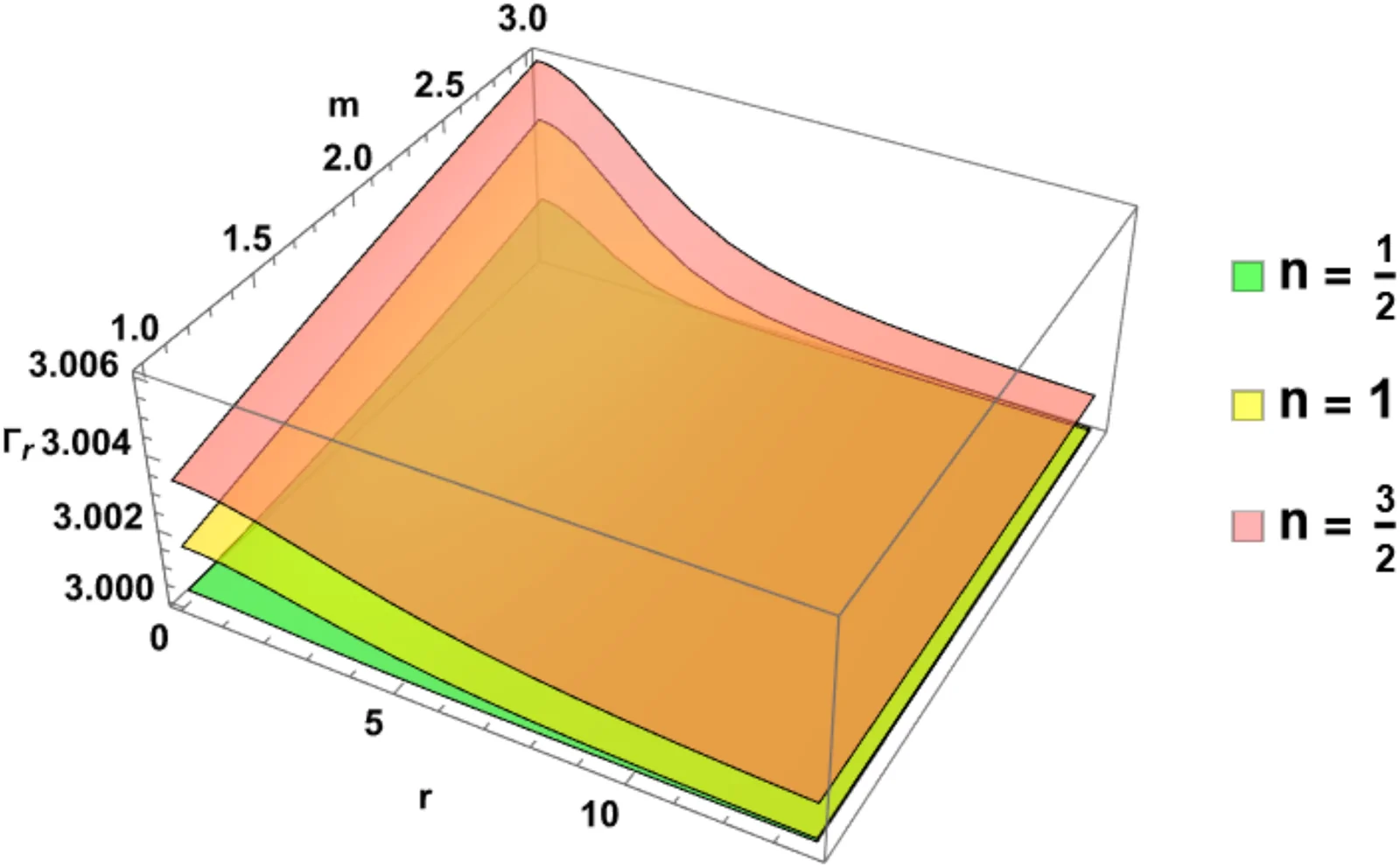
Radial profile of adiabatic index $\Gamma_{r}$ for PSR J0952–0607, remaining above the critical value 4/3 and confirming stability for different polytropic indices *n.*

**Fig 12:**
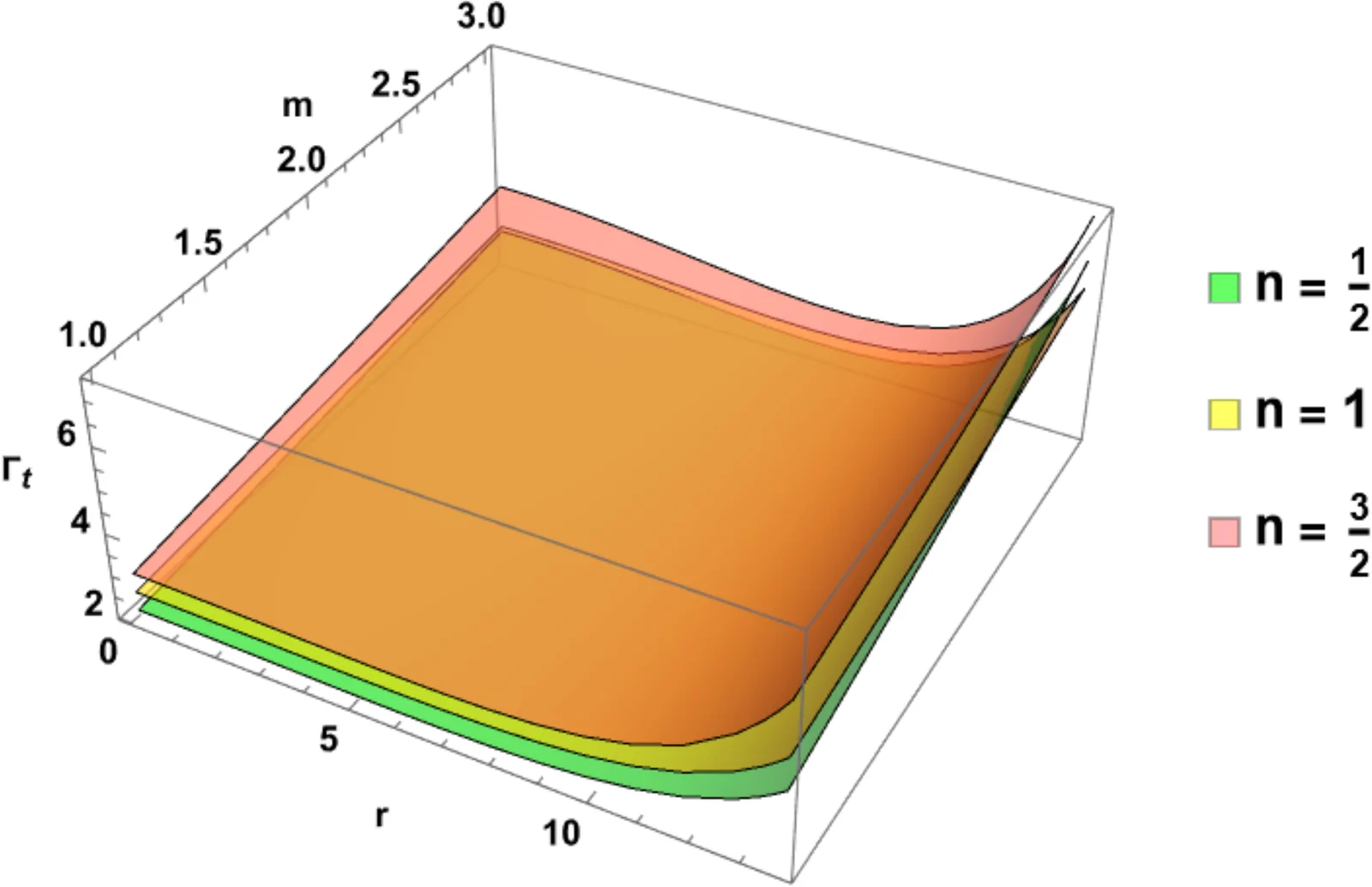
Tangential variation of the adiabatic index $\Gamma_{t}$ for PSR J0952–0607, remaining above the critical value 4/3 and confirming stability for different polytropic indices *n.*

Fig 11 illustrate the behavior of the radial $\Gamma_{r}$ adiabatic index showing that $\Gamma_{r}$ remain above the critical threshold of $\frac{4}{3}$ throughout the interior of PSR J0952-0607. This confirms that the proposed model satisfies the criterion for dynamical stability.

Fig 12 shows the behavior of the transverse $\Gamma_{t}$ adiabatic index which is also remain above the critical threshold of $\frac{4}{3}$ throughout the interior of PSR J0952-0607. This confirms that the proposed model satisfies the criterion for dynamical stability.

### 5.4 Energy conditions

Energy conditions refer to a set of physical constraints used to examine the nature of matter, whether ordinary or exotic within astrophysical compact objects. These criteria play a crucial role in assessing the robustness of theoretical models which can be examined through appropriate energy condition inequalities. The following summarizes and shows the Null (NEC), Weak (${\text{WEC}}_{r}$, ${\text{WEC}}_{t}$), Strong, Dominant (${\text{DEC}}_{r}$, ${\text{DEC}}_{t}$) and Trace (TEC) energy conditions with radial and tangential direction [66,67] that are pertinent to this investigation presented in Figs 13–18.

**Fig 13:**
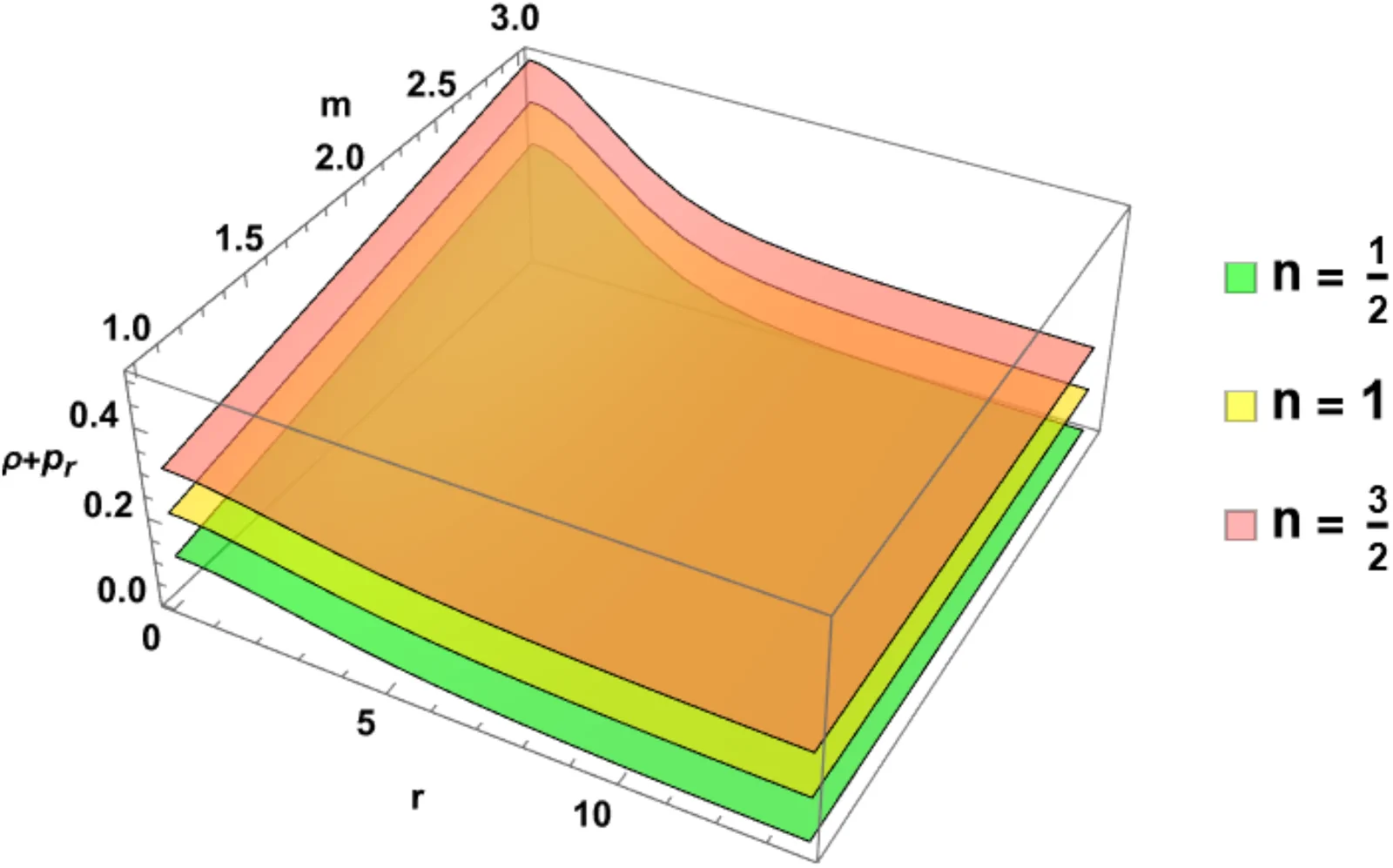
Radial variation of ${WEC}_{r}$ (*km*^–2^) for PSR J0952–060.

**Fig 14:**
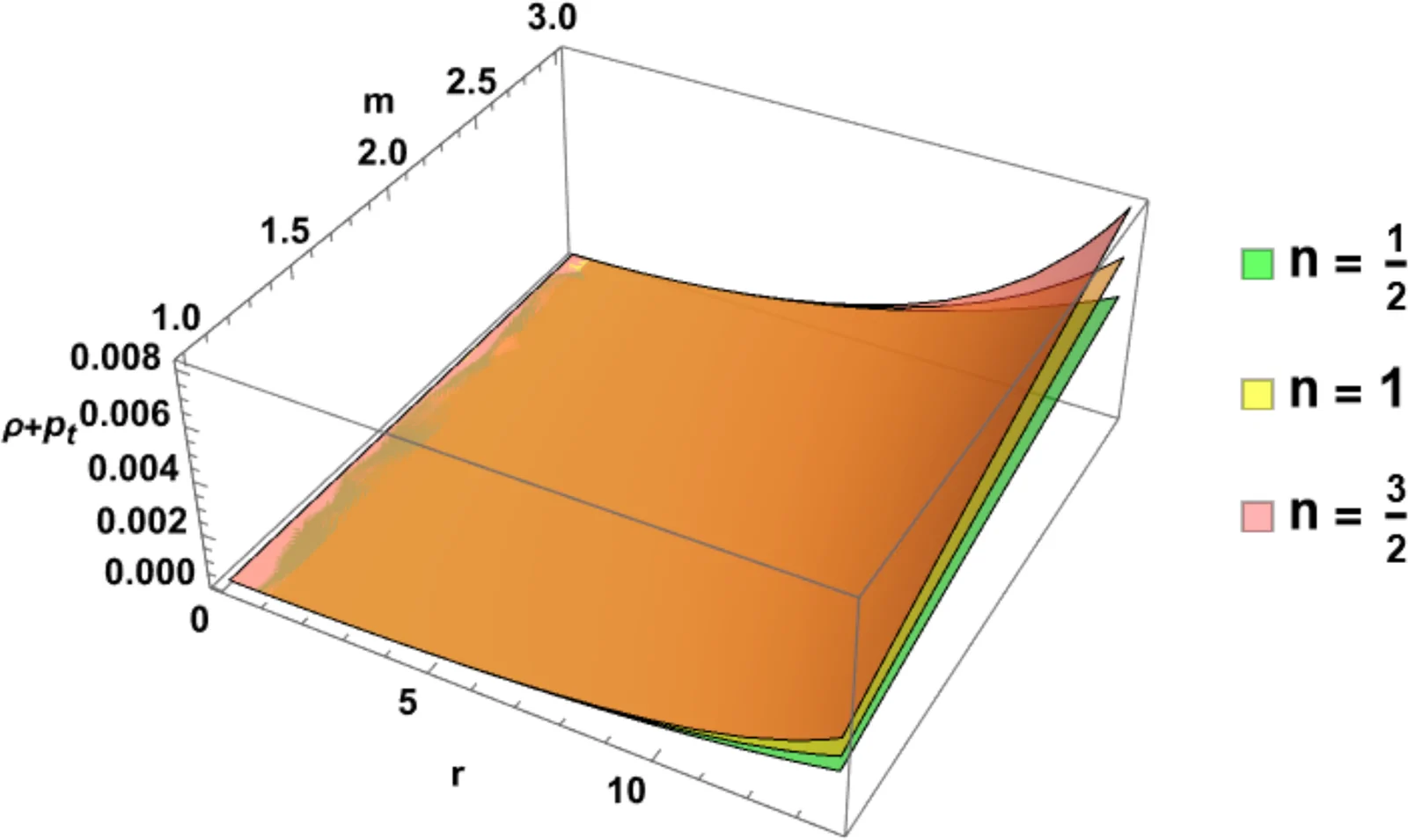
Radial variation of ${WEC}_{t}$ (*km*^–2^) for PSR J0952–0607.

**Fig 15:**
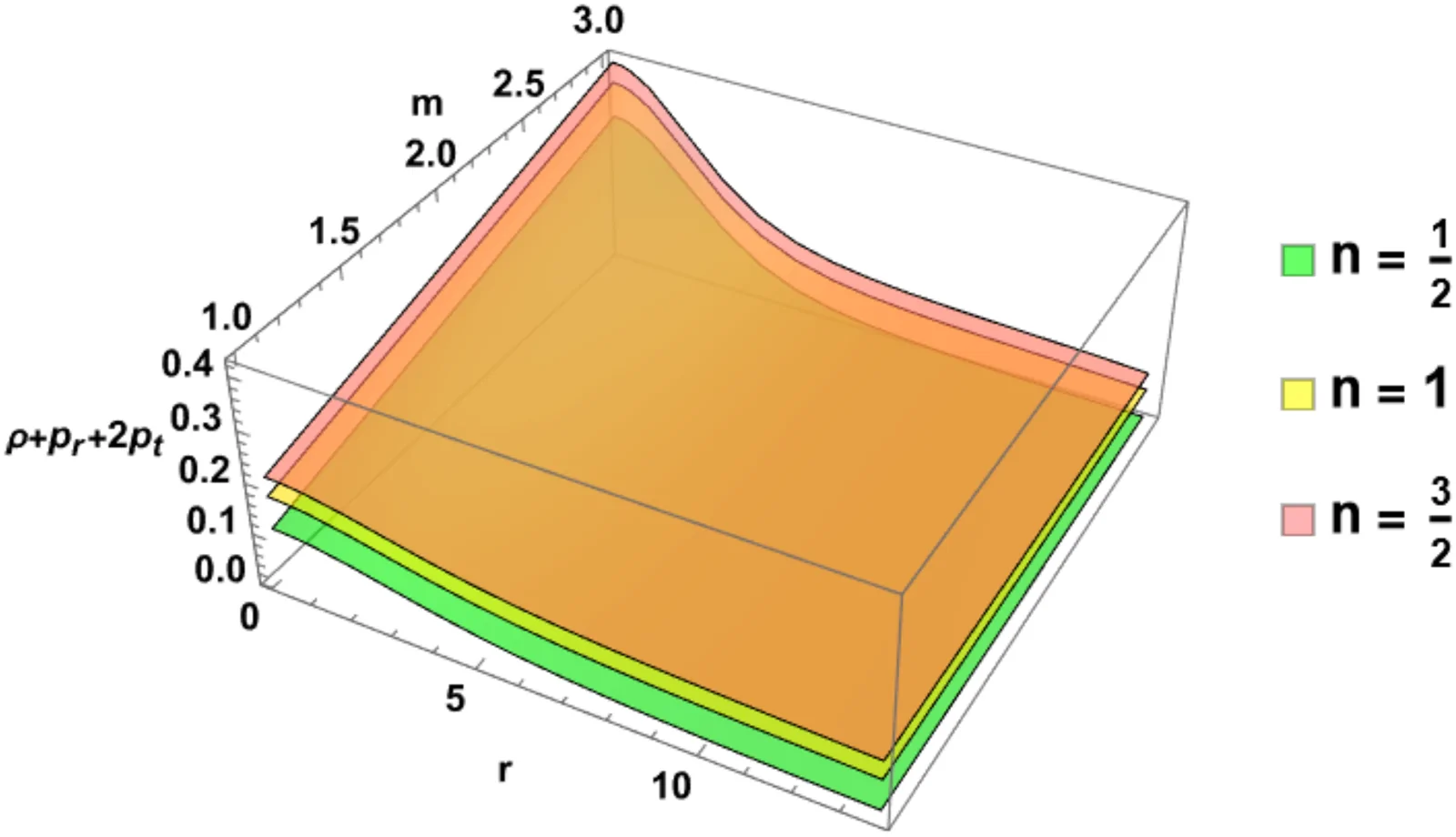
Radial profile of SEC (*km*^–2^) for PSR J0952–0607.

**Fig 16:**
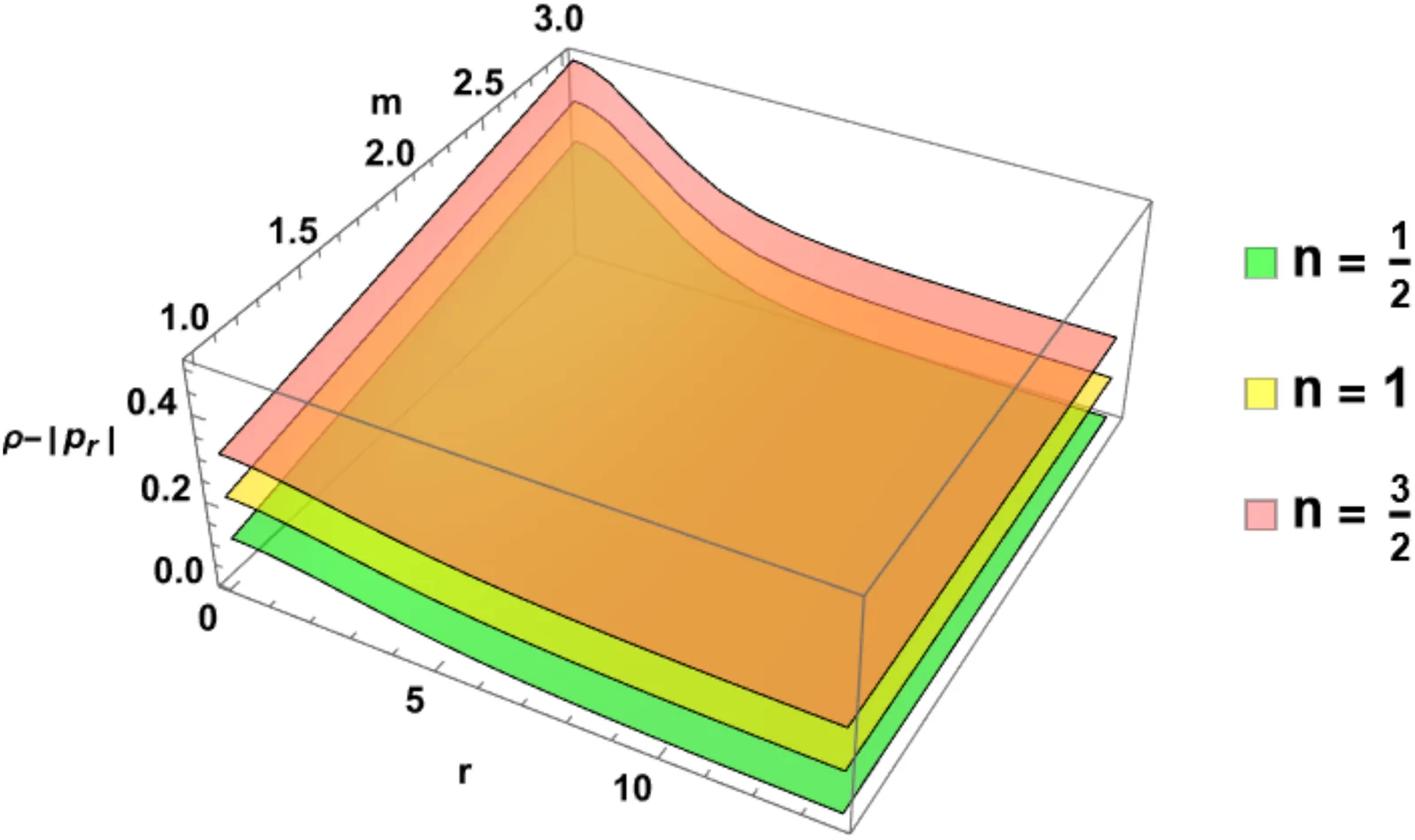
Radial variation of ${DEC}_{r}$ (*km*^–2^) for PSR J0952–0607.

**Fig 17:**
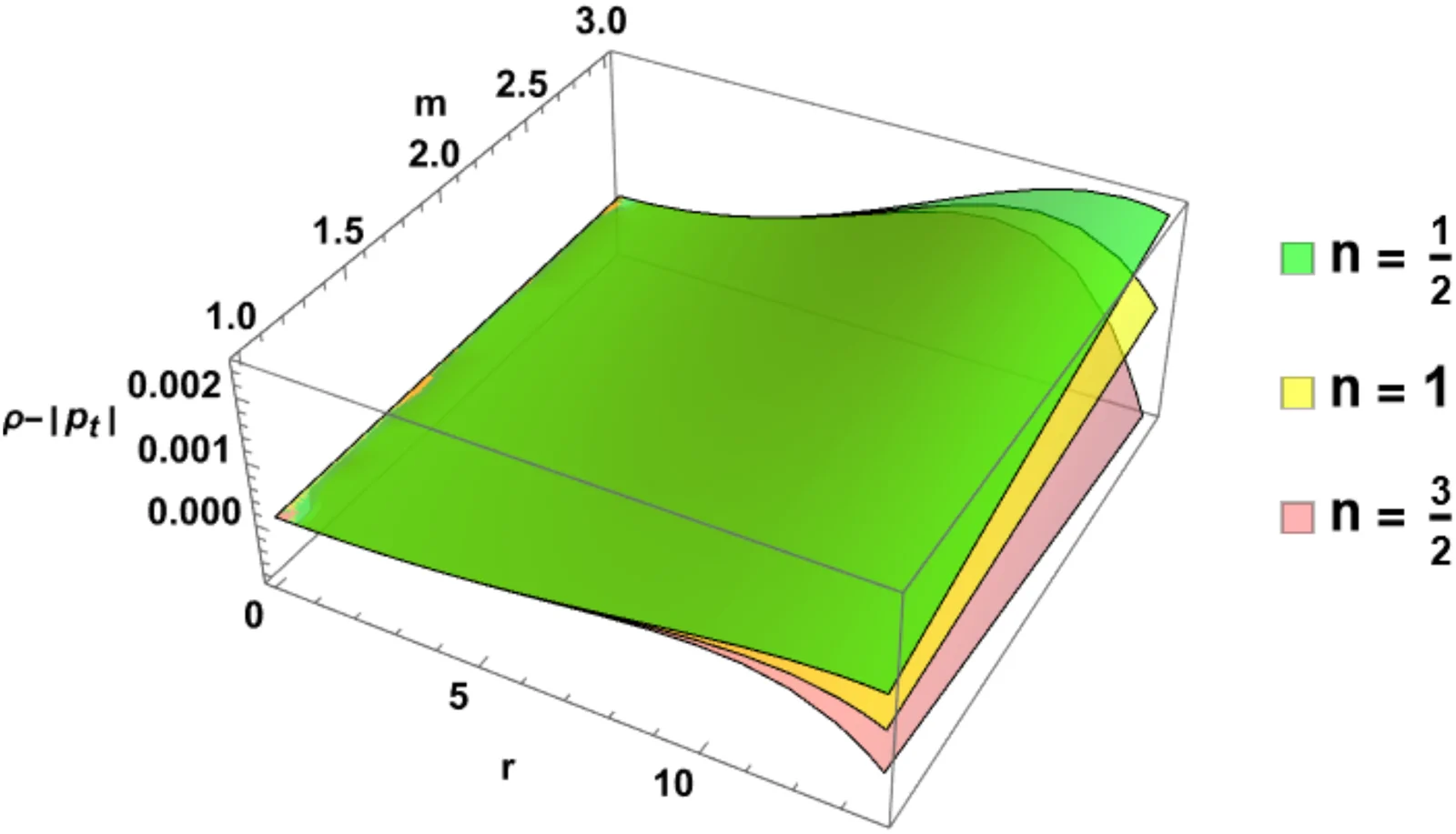
Radial profile of ${DEC}_{t}$ (*km*^–2^) for PSR J0952–0607.

**Fig 18:**
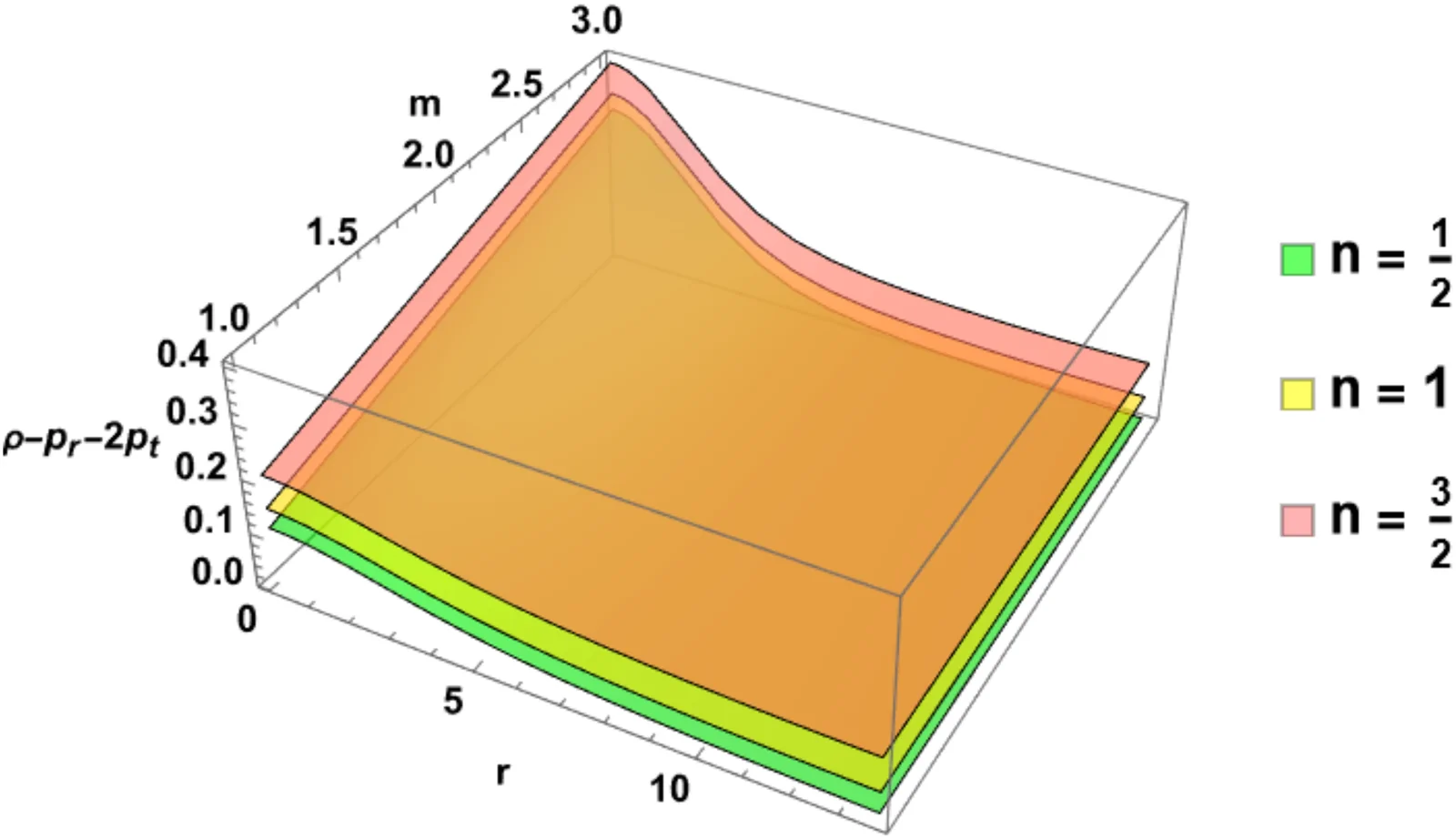
Radial variation of TEC (*km*^–2^) for PSR J0952–0607.

- NEC: (ρ≥0)
- TEC: ($\rho -p_{r}-2p_{t})\geq 0$
- SEC: ($\rho +p_{r}+2p_{t})\geq 0$
- ${\text{WEC}}_{r}$, ${\text{WEC}}_{t}$: ($\rho +p_{r}\geq 0$), ($\rho +p_{t}\geq 0$)
- ${\text{DEC}}_{r}$, ${\text{DEC}}_{t}$: ($\rho -|p_{r}|\geq 0$), $\rho -|p_{t}|\geq 0$

The NEC guaranties that ρ measured by a null (light-like) observer remains positive.

The behavior of ${\text{WEC}}_{r}$, defined by $\rho +p_{r}\geq 0$, remains positive throughout the stellar interior, as shown in Fig 13. This confirms that the radial pressure satisfies the weak energy condition over the entire configuration.

Similarly, ${\text{WEC}}_{t}$, given by $\rho +p_{t}\geq 0$, is satisfied at all radial points inside the star, as illustrated in Fig 14. This indicates that the tangential pressure also remains physically acceptable throughout the stellar structure.

Furthermore, the SEC surface, illustrated in Fig 15, ensures that the combined contribution of pressure and energy density strengthens the gravitational field, thus preserving the attractive nature of gravity.

Fig 16 presents the behavior of ${\text{DEC}}_{r}$, defined by $\rho -p_{r}\geq 0$, for PSR J0952-0607 as a three-dimensional surface with respect to the polytropic index $n=\frac{1}{2},\;1,\;\frac{3}{2}$ and the GTK metric parameter *m*. The positive values throughout the stellar interior confirm that the radial dominant energy condition is satisfied for all considered parameter choices.

Fig 17 illustrates ${\text{DEC}}_{t}$, given by $\rho -p_{t}\geq 0$, for the same range of the polytropic index *n* and GTK metric parameter *m*. Its positive behavior across the entire stellar configuration verifies that the tangential dominant energy condition remains fulfilled throughout the star.

Fig 18 illustrates the radial behavior of the TEC for PSR J0952–0607, represented as a three-dimensional surface for the polytropic index $n=\frac{1}{2},\;1,\;\frac{3}{2}$ and the GTK metric parameter *m*. Across the entire stellar interior, the TEC remains positive for all combinations of *n* and *m*, demonstrating that this condition is consistently satisfied.

### 5.5 Hydrostatic equilibrium (TOV) equation

The stellar objects require hydrostatic equilibrium to maintain its physical stability and consistency. The viability of massive pulsar PSR J0952-0607 under various forces is investigated using the principle of hydrostatic balance. The TOV relation given in (16) presents the stability condition for the anisotropic relativistic star configuration. This equation balances three fundamental forces of star such as the hydrostatic force $\left(F_{h}\right)$, the anisotropic force $\left(F_{a}\right)$, and the gravitational force $\left(F_{g}\right)$. The analysis of TOV equation is essential for verifying the stability of the stellar configuration throughout its interior [68]. The TOV equation is expressed in Eq. (16). The stability of the system is governed by Eq. (16), which incorporates the contributions of the hydrostatic, anisotropic, and gravitational forces $F_{h}$, $F_{a}$, and $F_{g}$ respectively.


$$\begin{array}{cc} F_{g} & =\frac{-\nu^{\prime }}{2}(\rho +p_{r}), \end{array}$$ (35)

$$\begin{array}{cc} F_{a} & =\frac{2\Delta }{r}, \end{array}$$ (36)

$$\begin{array}{cc} F_{h} & =-\frac{dp_{r}}{dr}, \end{array}$$ (37)

and


$$\begin{array}{c} F_{g}+F_{h}+F_{a}=0. \end{array}$$ (38)

Here, $F_{g}$ is the gravitational force that acts inward and tends to compress the stellar matter. The hydrostatic force $F_{h}$ originates from the radial-pressure gradient and generally acts outward to oppose the gravitational collapse. The anisotropic force $F_{a}$ arises from the difference between the tangential and radial pressures. For $\Delta =p_{t}-p_{r}>0$, it acts outward and provides additional support against gravity, whereas for Δ<0, it acts inward and enhances the gravitational contraction. Hydrostatic equilibrium is achieved when these three forces cancel each other at every point within the stellar interior.

Figs 19–21 illustrate the radial behavior of the forces $F_{h}$, $F_{g}$, and $F_{a}$ for $n=\frac{1}{2}$, 1, and 3/2. The profiles show that $F_{g}$ effectively counterbalances the collective effects of $F_{a}$ and $F_{h}$, thus supporting the equilibrium and stability of PSR J0952-0607.

**Fig 19:**
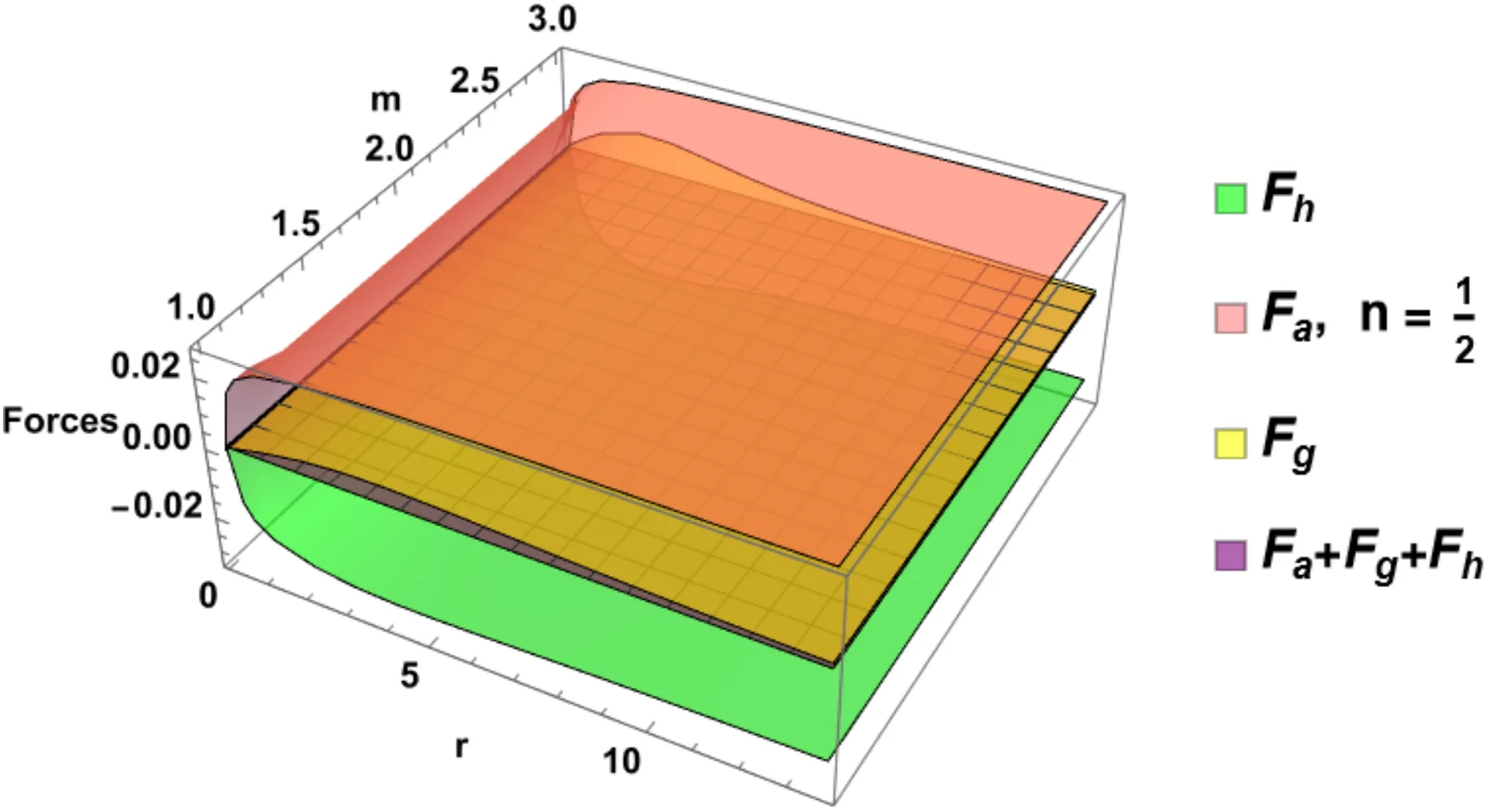
Radial variation of hydrostatic equilibrium forces for PSR J0952–0607, showing the balance among $F_{h}$, $F_{g}$, and $F_{a}$ forces.

**Fig 20:**
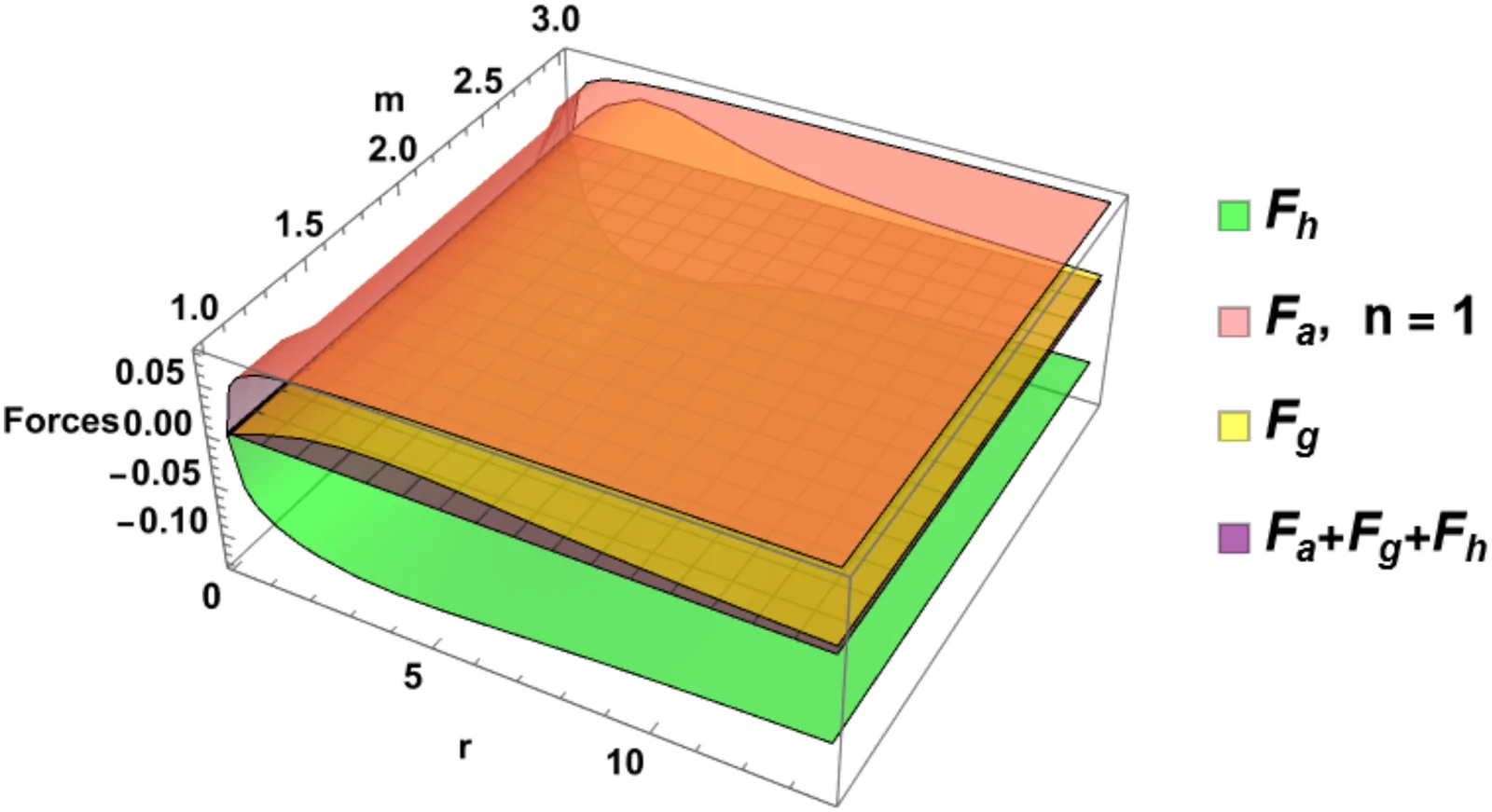
Radial variation of hydrostatic equilibrium forces for PSR J0952–0607, showing the balance among $F_{h}$, $F_{g}$, and $F_{a}$ forces.

**Fig 21:**
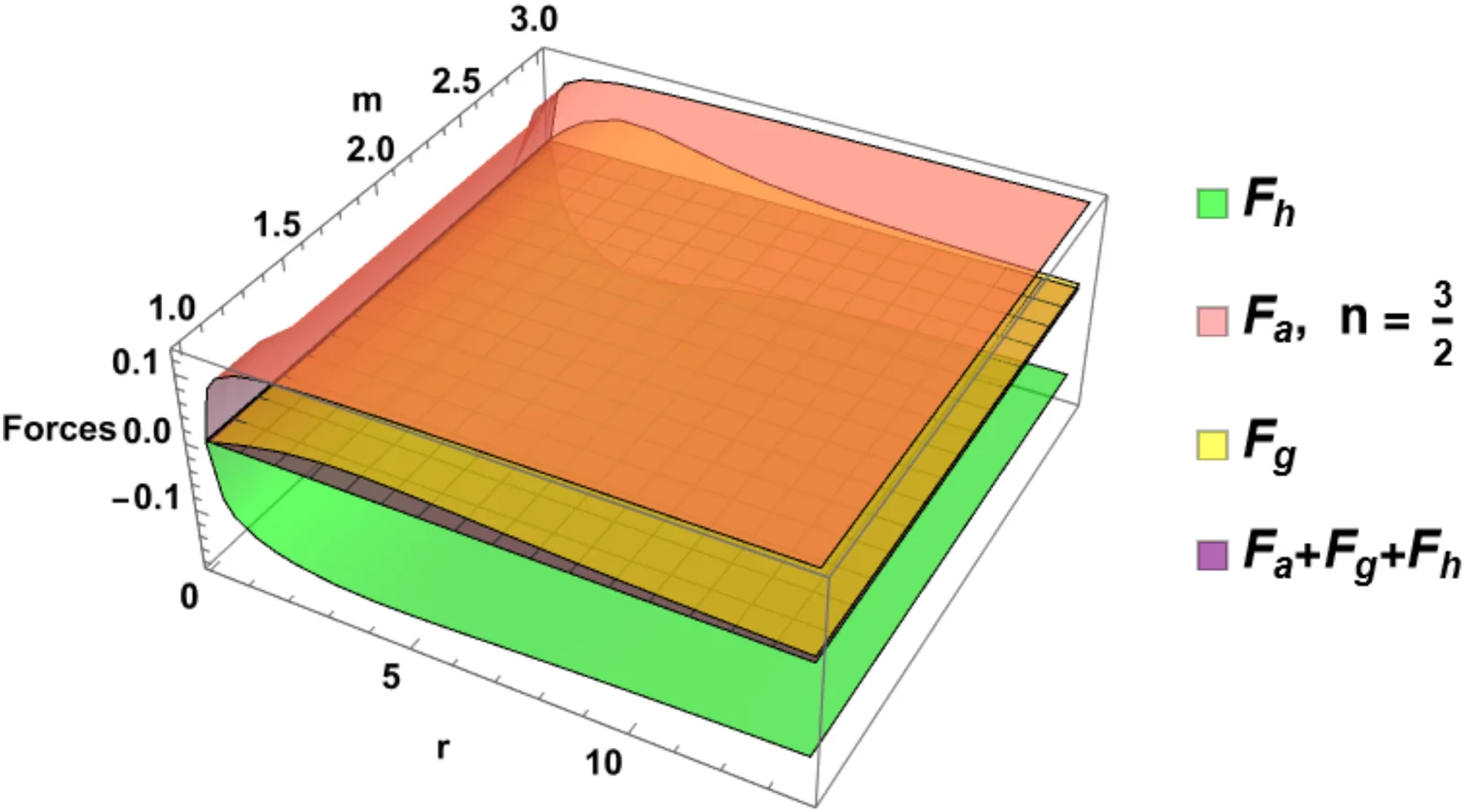
Radial variation of hydrostatic equilibrium forces for PSR J0952–0607, showing the balance among $F_{h}$, $F_{g}$, and $F_{a}$ forces.

### 5.6 Stellar mass and compactness

The mass function is described in Eq. (10). This function characterizes the radial distribution of the enclosed mass within PSR J0952-0607. The compactness *u*(*r*) is a function that gives the relationship between the enclosed mass and the radial distance *r*. It provides useful information on the internal compactness of the star and the strength of its gravitational field.

The *u*(*r*) is given as


$$\begin{array}{c} u(r)=\frac{m^{*}(r)}{r}. \end{array}$$ (39)

Here, $m^{*}(r)$ is given in Eq. (10). The reduction of smoothness to compactness is proportional to *m* as the star moves to the center of the star and towards the boundary surface, suggesting that the mass distribution is regular and physically acceptable. Greater values of *m* produce greater compactness at a given radius, corresponding to a greater gravitational field to increase the metric exponent. The fact that the interior behavior of *u*(*r*) is smooth and bounded confirms that there is no irregular behavior and supports the physical plausibility of the stellar model.

As illustrated in Fig 22, mass constantly grows with the radial distance indicating that there is a gradual accumulation of matter since the core to the outer limits of the star.

**Fig 22:**
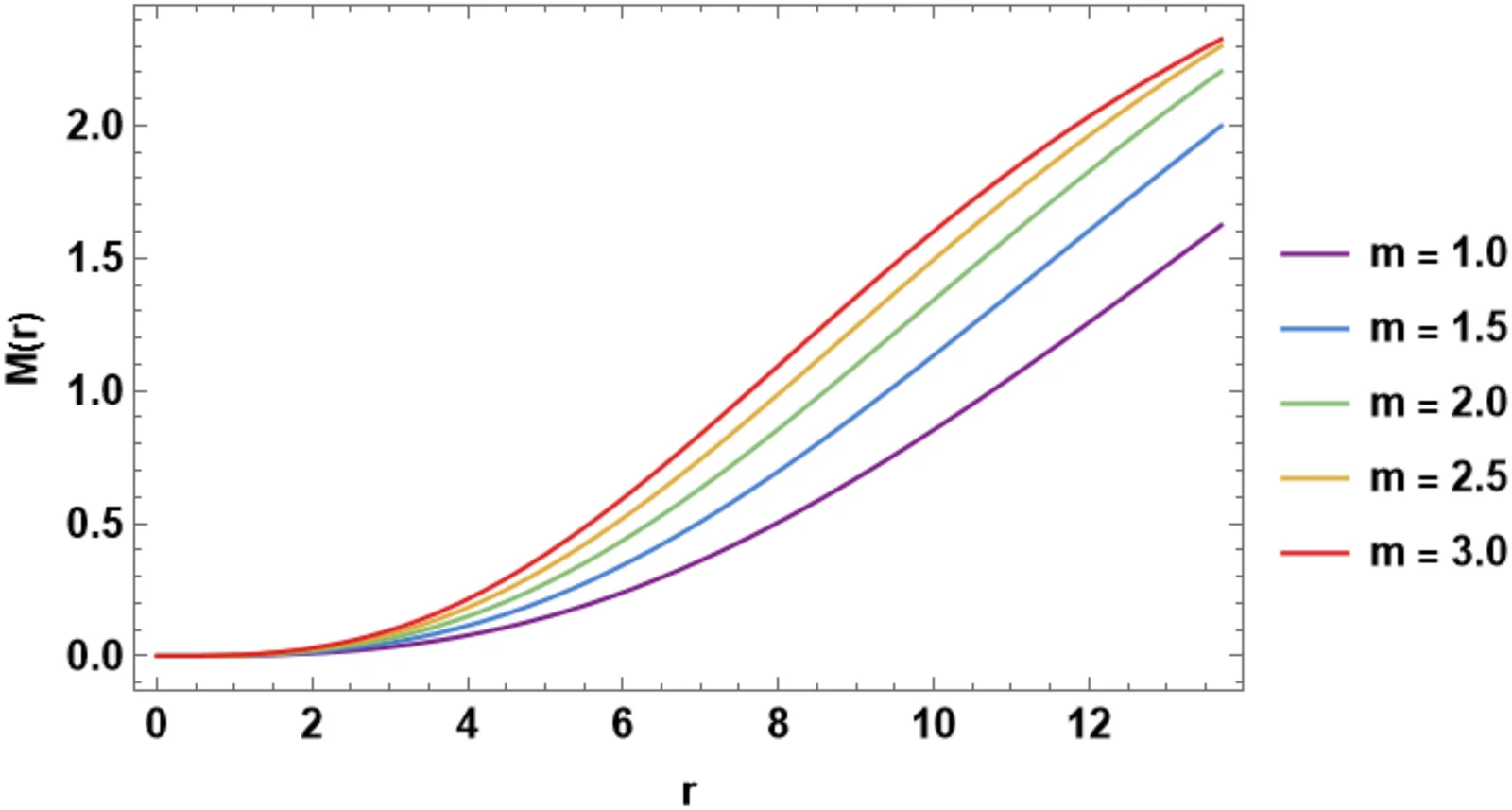
Radial variation of the enclosed mass *M*(*r*) for PSR J0952–0607, increasing smoothly with higher values for different values of *m.*

Fig 23 illustrates the radial variation of the compactness function *u*(*r*) for PSR J0952-0607. The compactness increases smoothly from the center toward the stellar surface, indicating a physically consistent accumulation of mass within the star.

**Fig 23:**
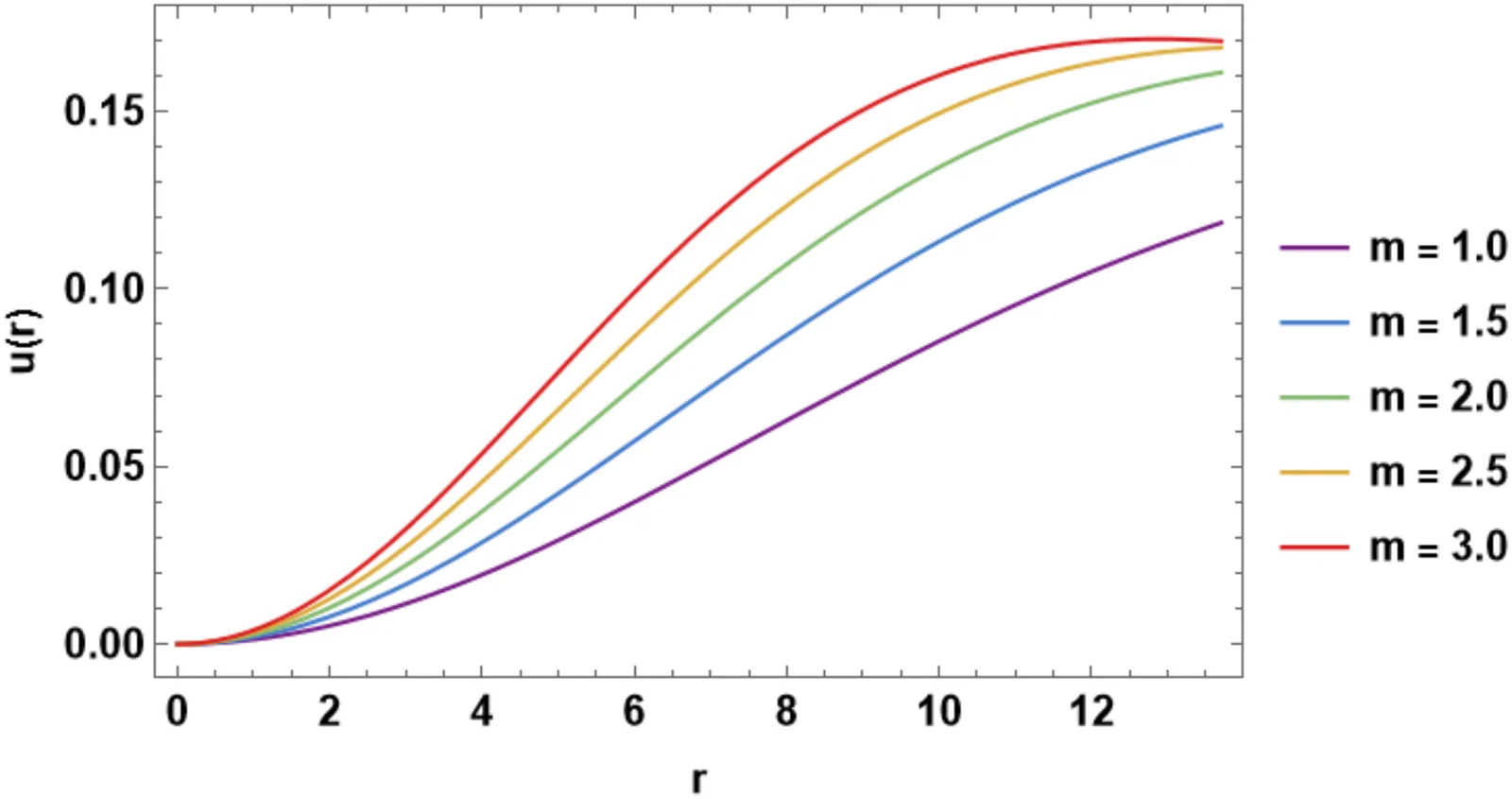
Radial variation of compactness factor *u*(*r*) for different values of *m.*

## 6. Conclusion

In this study, we investigated the internal structure and stability of a regular anisotropic compact object within the framework of GR. Assuming a static and spherically symmetric spacetime containing an anisotropic fluid distribution, exact stellar solutions are constructed by combining the GTK metric ansatz with a relativistic polytropic EOS. The physical viability of the interior configuration is examined by matching the GTK spacetime smoothly to the exterior Schwarzschild vacuum solution at the stellar surface. This matching condition is imposed through the continuity of the metric coefficients and their relevant derivatives, together with the condition that the radial pressure vanishes at the boundary.

To examine the astrophysical applicability of the model, we considered the ultra-massive pulsar PSR J0952–0607 with the measured mass $M=2.35\pm 0.17\;M_{⊙}$. Since a direct radius measurement of this source is not presently available, the value $R=13.7_{-1.5}^{+2.6}\;km$ is adopted as an observationally motivated representative radius based on the NICER and XMM–Newton measurement of the massive pulsar PSR J0740 + 6620. Therefore, the numerical results presented here should be understood as conditional on this adopted radius rather than as a source-specific radius determination for PSR J0952–0607.

The numerical and graphical analysis show that ρ, $p_{r}$, and $p_{t}$ remain finite at the stellar center and decrease smoothly toward the boundary, as illustrated in Figs 1, 3 and 4. The GTK exponent *m* influences the radial curvature and matter concentration through the metric potential $e^{\lambda (r)}=(1+ar^{2}+br^{4}{)}^{m}$, whereas the polytropic index *n* controls the pressure response through $p_{r}=\alpha \rho^{1+1/n}$. Accordingly, variations in *m* modify the central concentration and radial compactness of the configuration, while variations in *n* change the effective stiffness of the stellar matter. In the present analysis, the representative polytropic indices $n=\frac{1}{2},\;1,\;\frac{3}{2}$ were considered, together with the GTK range 1≤m≤3.

The anisotropy profile shown in Fig 5 vanishes at the center, as required by spherical symmetry, and becomes positive away from the core. Since $\Delta =p_{t}-p_{r}>0$, the corresponding anisotropic force acts outward and assists the radial pressure gradient in opposing the strong inward gravitational attraction. The gradients dρ/dr, $dp_{r}/dr$, and $dp_{t}/dr$, displayed in Figs 2, 6 and 7, remain negative throughout the stellar interior, indicating that the density and pressures decrease monotonically from the center toward the surface.

The radial and tangential sound speeds presented in Figs 8 and 9 remain within the causal interval, $0\leq v_{r}^{2}\leq 1,\;0\leq v_{t}^{2}\leq 1$, for the parameter values considered. The difference between the sound speeds, shown in Fig 10, remains bounded in magnitude by unity. The Herrera cracking condition in Fig 10 satisfies the condition of less than 1 within the stellar boundary. The radial and tangential adiabatic indices shown in Figs 11 and 12 remain above the critical value 4/3, indicating an adequate pressure response against infinitesimal compression within the considered parameter domain.

The standard null, weak, strong, and dominant energy conditions are also examined through the exact inequalities involving ρ, $p_{r}$, and $p_{t}$. The corresponding combinations displayed in Figs 13 – 18 remain non-negative for the admissible configurations, supporting the physical acceptability of the matter distribution. Hydrostatic equilibrium was analyzed through the generalized TOV equation, The force profiles in Figs 19 – 21, corresponding to $n=\frac{1}{2}$, 1, and $\frac{3}{2}$, show that the inward gravitational force is balanced by the hydrostatic and anisotropic contributions throughout the stellar interior.

Figs 22 and 23 present the enclosed mass *M*(*r*) and compactness *u*(*r*) as functions of the radial coordinate for different values of the GTK exponent *m*. Both quantities increase monotonically from the center and attain their maximum values at the stellar surface. Larger values of *m* generally produce higher enclosed mass and compactness within the selected parameter range, indicating a stronger concentration of matter and a more tightly bound configuration. These results are compatible with representative mass–radius constraints for massive neutron stars, but they should not be interpreted as a direct NICER validation of PSR J0952–0607 itself [6].

The present results indicate that the GTK geometry combined with a polytropic EoS can generate regular and physically admissible anisotropic configurations for ultra-massive compact stars. Nevertheless, the model has important limitations. The metric potentials are prescribed through a specific geometric ansatz rather than derived from the microscopic properties of dense matter. The polytropic EoS is phenomenological and does not explicitly include composition changes, phase transitions, superfluidity, strong magnetic fields, rapid rotation, or other microphysical effects expected inside realistic neutron stars. Moreover, the conclusions are restricted to the selected ranges of *m* and *n* and depend on the adopted stellar radius and polytropic parameter α. Therefore, the present configuration should be regarded as a viable phenomenological model, rather than a unique or definitive description of the internal structure of PSR J0952–0607.

Future investigations may extend this framework by incorporating realistic tabulated or piecewise equations of state, rotational and magnetic effects, and a wider parameter domain. A comparison with independent mass–radius, tidal-deformability, and moment-of-inertia constraints would provide a more stringent assessment of the astrophysical applicability of the model.

## Appendix

### Derivation of EFEs

The main intermediate steps leading to Eqs. (12)–(17). Let


$$\begin{array}{c} X(r)=1+ar^{2}+br^{4},\;Y(r)=a+2br^{2}, \end{array}$$ (40)

so that the GTK metric potentials become


$$\begin{array}{c} e^{\lambda }=X^{m},\;e^{-\lambda }=X^{-m},\;e^{\nu }=A^{2}e^{Br^{2}}. \end{array}$$ (41)

The required derivatives are


$$\begin{array}{c} X^{\prime }=2ar+4br^{3}=2rY, \end{array}$$ (42)

$$\begin{array}{c} \lambda^{\prime }=m\frac{X^{\prime }}{X}=\frac{2mrY}{X},\;\nu^{\prime }=2Br,\;\nu^{\prime \prime }=2B. \end{array}$$ (43)

Substitution of $e^{-\lambda }=X^{-m}$ and $\lambda^{\prime }=2mrY/X$ into the temporal component of the EFEs,


$$\begin{array}{c} 8\pi \rho =e^{-\lambda }\left(\frac{\lambda^{\prime }}{r}-\frac{1}{r^{2}}\right)+\frac{1}{r^{2}}, \end{array}$$ (44)

gives


$$\begin{array}{cc} 8\pi \rho & =X^{-m}\left(\frac{2mY}{X}-\frac{1}{r^{2}}\right)+\frac{1}{r^{2}}\\ & =\frac{1-X^{-m}}{r^{2}}+2mYX^{-m-1}. \end{array}$$ (45)

Therefore,


$$\begin{array}{c} \rho (r)=\frac{1}{8\pi }\left[\frac{1-X^{-m}}{r^{2}}+2mYX^{-m-1}\right]. \end{array}$$ (46)

If the convention 8π=1 is adopted, Eq. (46) reduces directly to the form used in Eq. (12).

The radial pressure is prescribed through the relativistic polytropic EoS


$$\begin{array}{c} p_{r}=\alpha \rho^{\gamma },\;\gamma =1+\frac{1}{n}. \end{array}$$ (47)

Using Eq. (46), we obtain


$$\begin{array}{c} p_{r}(r)=\alpha {\left\{\frac{1}{8\pi }\left[\frac{1-X^{-m}}{r^{2}}+2mYX^{-m-1}\right]\right\}}^{1+1/n}. \end{array}$$ (48)

The radial pressure gradient then follows from the chain rule as


$$\begin{array}{c} \frac{dp_{r}}{dr}=\alpha \left(1+\frac{1}{n}\right)\rho^{1/n}\frac{d\rho }{dr}. \end{array}$$ (49)

Differentiating Eq. (46) gives


$$\begin{array}{cc} 8\pi \frac{d\rho }{dr}= & -\frac{2}{r^{3}}+\frac{2X^{-m}}{r^{3}}+\frac{mX^{\prime }X^{-m-1}}{r^{2}}\\ & +8mbrX^{-m-1}-2m(m+1)YX^{\prime }X^{-m-2}. \end{array}$$ (50)

Since $X^{\prime }=2rY$, substitution of Eq. (50) into Eq. (49) yields the explicit expression reported for $dp_{r}/dr$.

The tangential pressure is not prescribed by an independent EoS. It follows from the angular component of the Einstein field equations,


$$\begin{array}{c} 8\pi p_{t}=\frac{e^{-\lambda }}{4}\left[\nu^{\prime 2}+2\nu^{\prime \prime }-\nu^{\prime }\lambda^{\prime }+\frac{2\nu^{\prime }}{r}-\frac{2\lambda^{\prime }}{r}\right]. \end{array}$$ (51)

Using the GTK derivatives, this becomes


$$\begin{array}{cc} 8\pi p_{t}= & \frac{X^{-m}}{4}\left[4B^{2}r^{2}+4B-\frac{4Bmr^{2}Y}{X}+4B-\frac{4mY}{X}\right]\\ = & X^{-m}\left[B^{2}r^{2}+2B-\frac{Bmr^{2}Y}{X}-\frac{mY}{X}\right]. \end{array}$$ (52)

Equivalently, the conservation equation


$$\begin{array}{c} \frac{dp_{r}}{dr}+\frac{\nu^{\prime }}{2}(\rho +p_{r})-\frac{2}{r}(p_{t}-p_{r})=0 \end{array}$$ (53)

gives


$$\begin{array}{c} p_{t}=p_{r}+\frac{r}{2}\frac{dp_{r}}{dr}+\frac{r\nu^{\prime }}{4}(\rho +p_{r}). \end{array}$$ (54)

Since $\nu^{\prime }=2Br$, Eq. (54) may also be written as


$$\begin{array}{c} p_{t}=p_{r}+\frac{r}{2}\frac{dp_{r}}{dr}+\frac{Br^{2}}{2}(\rho +p_{r}). \end{array}$$ (55)

Finally, the anisotropy is obtained as


$$\begin{array}{c} \Delta =p_{t}-p_{r}=\frac{r}{2}\frac{dp_{r}}{dr}+\frac{r\nu^{\prime }}{4}(\rho +p_{r}). \end{array}$$ (56)

Thus, ρ is fixed by the temporal field equation, $p_{r}$ by the polytropic EoS, and $p_{t}$ and Δ by the angular field equation or, equivalently, the conservation equation.

### Derivation of boundary conditions

At the boundary *r* = *R*, the interior GTK spacetime is matched smoothly to the exterior Schwarzschild solution,


$$\begin{array}{c} ds^{2}=\left(1-\frac{2M}{r}\right)dt^{2}-{\left(1-\frac{2M}{r}\right)}^{-1}dr^{2}-r^{2}d\Omega^{2}. \end{array}$$ (57)

The interior metric is


$$\begin{array}{c} ds^{2}=A^{2}e^{Br^{2}}dt^{2}-{\left(1+ar^{2}+br^{4}\right)}^{m}dr^{2}-r^{2}d\Omega^{2}. \end{array}$$ (58)

Continuity of the temporal metric component at *r* = *R* gives


$$\begin{array}{c} A^{2}e^{BR^{2}}=1-\frac{2M}{R}. \end{array}$$ (59)

Similarly, continuity of the radial metric component gives


$$\begin{array}{c} {\left(1+aR^{2}+bR^{4}\right)}^{m}={\left(1-\frac{2M}{R}\right)}^{-1}. \end{array}$$ (60)

The derivative of the interior temporal component is


$$\begin{array}{c} \frac{d}{dr}\left(A^{2}e^{Br^{2}}\right)=2BrA^{2}e^{Br^{2}}, \end{array}$$ (61)

whereas the derivative of the Schwarzschild temporal component is


$$\begin{array}{c} \frac{d}{dr}\left(1-\frac{2M}{r}\right)=\frac{2M}{r^{2}}. \end{array}$$ (62)

Therefore, continuity of the first derivative of $g_{tt}$ at the boundary requires


$$\begin{array}{c} 2BRA^{2}e^{BR^{2}}=\frac{2M}{R^{2}}. \end{array}$$ (63)

Using Eq. (59) in Eq. (63), we obtain


$$\begin{array}{c} 2BR\left(1-\frac{2M}{R}\right)=\frac{2M}{R^{2}}, \end{array}$$ (64)

and hence


$$\begin{array}{c} B=\frac{M}{R^{3}\left(1-\frac{2M}{R}\right)}=\frac{M}{R^{2}(R-2M)}. \end{array}$$ (65)

From Eq. (59), the constant *A* is obtained as


$$\begin{array}{c} A={\left(1-\frac{2M}{R}\right)}^{1/2}\exp\left(-\frac{BR^{2}}{2}\right). \end{array}$$ (66)
